# Torque-speed relationship of the flagellar motor with dual-stator systems in *Pseudomonas aeruginosa*

**DOI:** 10.1128/mbio.00745-24

**Published:** 2024-10-30

**Authors:** Haolin Wu, Zhengyu Wu, Maojin Tian, Rongjing Zhang, Junhua Yuan

**Affiliations:** 1 Hefei National Research Center for Physical Sciences at the Microscale and Department of Physics, University of Science and Technology of China, Hefei, Anhui, China; 2 Center of Translational Medicine, Central Hospital Affiliated to Shandong First Medical University, Jinan, Shandong, China; 3 Center of Translational Medicine, Zibo Central Hospital Affiliated to Binzhou Medical University, Zibo, Shandong, China; University of Utah, Salt Lake, Utah, USA

**Keywords:** optical tweezers, bacterial motility, bacterial flagellar motor, load dependence, slip bond, catch bond

## Abstract

The single polar flagellar motor in *Pseudomonas aeruginosa* is equipped with two stator systems, MotAB and MotCD, both driven by H^+^ ions. The torque-speed relationship for flagellar motors with two stator systems has not been explored previously. In this study, we developed a method that utilizes optical trapping and fluorescence labeling to measure the torque-speed relationships for the wild-type *P. aeruginosa* motor with dual stators and mutant strains with a single stator system, revealing surprising differences in them. Moreover, we found that the MotAB stators exhibit slip-bond behavior in load dependence, contrasting with the catch-bond behavior of the MotCD stators and *Escherichia coli* stators. Further examination of the solvent isotope and pH effects on the torque-speed relationships of these stator systems provided additional insights into their dynamics. Interestingly, we discovered that the torque of the wild-type motor is similar to the combined torque of motors with MotAB or MotCD stators, indicating an additive contribution from the two stator types in the wild-type motors. These findings underscore the enhanced adaptability of *P. aeruginosa* to a wide range of external environments with varying load conditions.

**IMPORTANCE:**

We developed a novel method to measure the flagellar motor torque-speed relationship by trapping a swimming bacterium using optical tweezers. Using the *P. aeruginosa* flagellar motor as a model system to investigate motor dynamics with dual stator types, we measured the torque-speed relationships for wild-type motors with dual stator types and mutants with a single type. We found drastic differences that stem from the varying load dependencies of stator stability. These variations enable bacteria to rapidly adjust their stator composition in response to external load conditions. Interestingly, we observed that the torque of the wild-type motor is akin to the cumulative torque of motors with either stator type, indicating an additive contribution from the two stator types in wild-type motors. The methodology we established here can be readily employed to study motor dynamics in other flagellated bacteria.

## INTRODUCTION

Many species of flagellated bacteria swim by rotating their flagella, each driven by a rotary motor embedded in the cell envelope (1–3). The construction of these flagellar motors involves approximately 20 types of proteins (4, 5). These motors share many homologous features across different flagellated bacterial species, including a membrane-embedded rotor complex and a number of torque-generating stator units. The flagellar motors are driven by the transmembrane ion flux (6, 7), such as H^+^ for most neutrophils (8, 9) and Na^+^ for the alkalophiles and marine *Vibrio* species (10–12). When the ions flow across the ion channels formed across the stator units, the conformational changes of stator units result in torque generation (13–15). Different bacterial species have different types of stator units. For example, *Escherichia coli* and *Salmonella* species have only one stator system, H^+^-powered MotAB stators (8, 16). However, some bacteria have two stator systems driven by two types of ions, such as H^+^-powered MotAB and Na^+^-powered PomAB (for *Shewanella oneidensis*)/MotPS (for *Bacillus subtilis*) stators (17, 18). The mechanism of cell movement driven by dual ions can improve the adaptability of bacteria to the diversity of the external environments.

Unlike the flagellar bacteria species mentioned above, the single polar flagellum of *Pseudomonas aeruginosa* PAO1 possesses two stator systems driven only by H^+^, MotAB (PA4953/PA4954), and MotCD (PA1460/PA1461). Each of these systems can independently enable cell swimming (19, 20). Previous studies found that the combination of MotAB and MotCD stators can provide stable motor torque and maintain a consistent motor speed under various load conditions in liquid environments of various viscosities (21). Notably, the MotCD stator can propel bacterial cells swimming in high-load conditions, such as in liquid environments with extremely high viscosity or during swarming on agar surfaces (19, 22). This suggests that the dual H^+^-powered stator systems of *P. aeruginosa* play different roles under different load conditions and movement modes. However, the dynamics of torque generation for each set of stators and the function of the dual-stator systems under various loads remain unclear. To investigate this, we aim to measure the torque-speed relationship of the two stator systems under a wide range of load conditions (23, 24).

Previous studies had measured the torque-speed curves for *E. coli* (25–27) and *Vibrio alginolyticus* (28) using the bead assay. These studies found two dynamic regimes of motor function: one at lower speeds, where the torque remained approximately constant, and another at higher speeds, where the torque dropped rapidly to zero. It would be interesting to see whether the dual-stator systems of *P. aeruginosa* behave differently and how the two types of stators contribute to the motor output.

Here, using optical tweezers, we measured the torque-speed relationship of the wild-type *P. aeruginosa* motor with dual-stator systems and mutant strains with a single stator system. We discovered surprising differences in their torque-speed relationships. Further investigations revealed slip-bond behavior in the load dependence of MotAB stators, which contrasts with the catch-bond behavior of the MotCD stators and *E. coli* MotAB stators (21, 29). Our examination of the solvent-isotope and pH effects on the torque-speed relationships of these stator systems provided further insights into their dynamics.

## RESULTS

### Measuring the torque-speed relationship using optical tweezers and fluorescence labeling

We previously developed a bead assay to study the motor dynamics in *P. aeruginosa*. In this assay, a 1-μm-diameter streptavidin-modified latex bead was attached to a biotinylated flagellar filament stub and used as an indicator of motor rotation (21). To measure the torque-speed relationship, we could label a 0.3-μm-diameter bead to the filament stub (a low-load condition) and use media with different concentrations of Ficoll to change the load conditions. However, the efficiency of labeling the smaller bead was extremely low, likely due to the significantly fewer number of streptavidin sites on the bead. Thus, we developed a new method using optical trapping to measure the torque-speed relationship.

**Fig 1:**
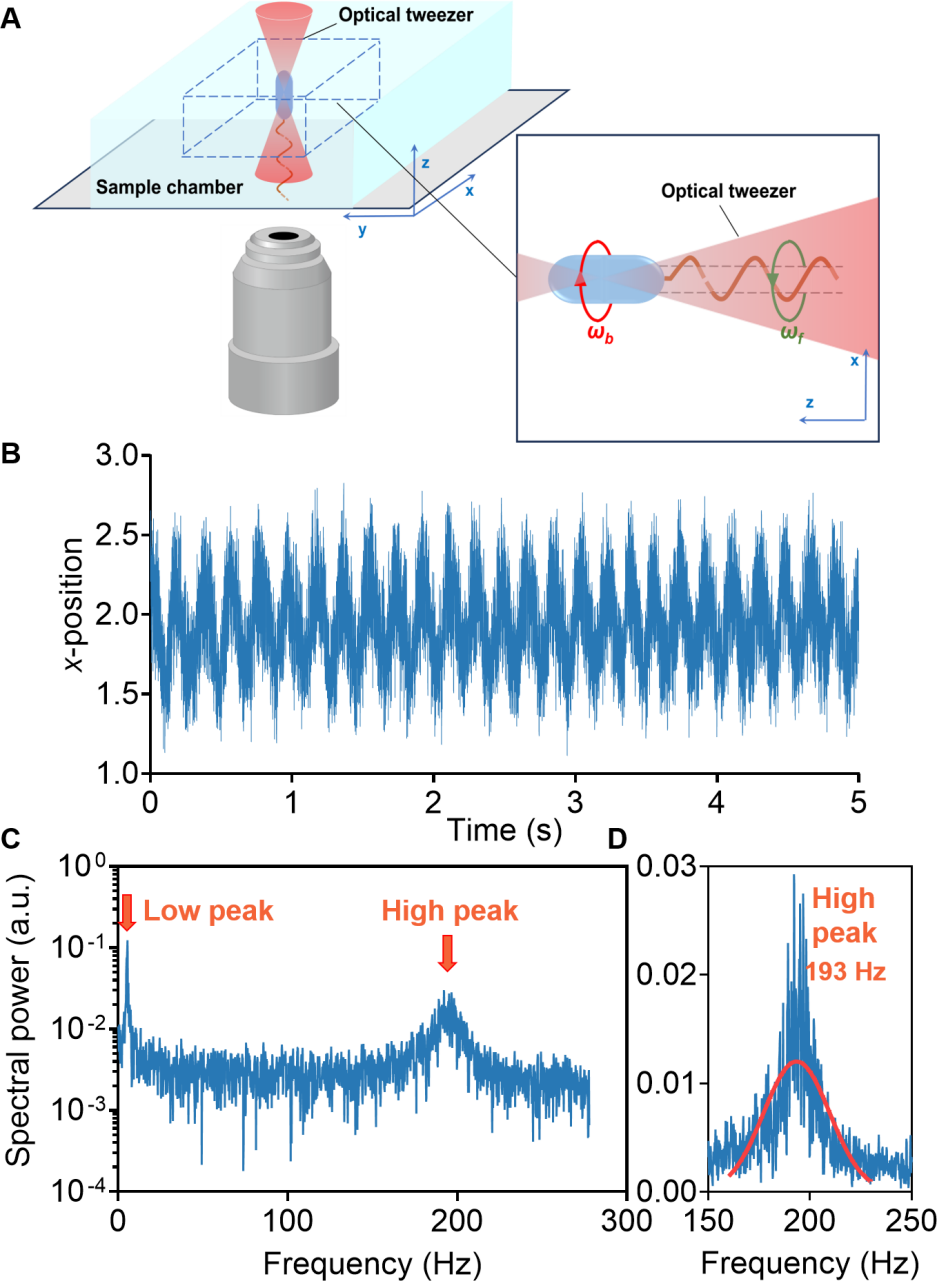
(**A**) Experimental setup and cell configuration. The cell body was trapped along the *z*-direction by an optical trap and rotated oppositely to the flagellar rotation. (**B**) A typical optical trap signal of a Δ*motAB* mutant cell along the *x*-direction in motility buffer. (**C**) The power spectrum of the signal is shown in (**B**), with the two peaks representing the rotation speeds of the cell body (low peak) and flagellum (high peak), respectively. (**D**) A zoom-in view of the high peak in (**C**). The red solid line is a Gaussian fit, obtaining the peak position of 193 Hz.

The idea of the method is as follows. *P. aeruginosa* is singly flagellated, with the only flagellar motor located at the cell pole for swimming (30). Under an external torque-free condition, the motor torque drives the counter-rotation of the cell body and the flagellar filament, and the motor speed is the sum of the rotation speeds of the cell body and flagellar filament. We can measure the rotation speeds of the cell body and flagellar filament by trapping the swimming bacterium using an optical tweezer (Fig. 1A). In addition, we can determine the drag coefficient of the flagellar filament through fluorescence labeling. We can obtain the geometric parameters of the flagellar filament by visualization, such as filament helix radius, contour length, and pitch, and then calculate the drag coefficient using these parameters. The details of this calculation are shown in Materials and Methods. The motor torque can then be calculated by multiplying the drag coefficient by the flagellar rotation speed. As the flagellar motor driving the rotation of a filament in water is under low load (31), we can construct the torque-speed curve by increasing the medium viscosity with different concentrations of Ficoll.

The experimental setup and operation details are presented in the Materials and Methods section. The cell body, although trapped and immobilized by the optical tweezer, exhibited motile behavior. This was detected by projecting light from the trapping beam onto a position-sensitive photodetector. The output of this photodetector, known as the optical trap signal, was recorded at a sampling rate of 5,000 Hz for rotation speeds below 300 Hz, and 10,000 Hz for rotation speeds above 300 Hz. The rotational frequencies of flagellar filament ***ω_f_*** and cell body ***ω_b_*** were obtained by analyzing the power spectrum of the signal. Figure 1B illustrates a typical optical trap signal of a motile cell along the *x*-axis, and its corresponding power spectrum, calculated *via* Fourier transform, is shown in Fig. 1C. The power spectrum displayed two peaks, representing the rotation speeds of the cell body $\omega_{b}$ (low peak) and the flagellum $\omega_{f}$ (high peak), respectively, and the flagellar motor rotation speed $\omega_{motor}$ is the sum of $\omega_{b}$ and $\omega_{f}$.

To assess potential photodamage from the optical trap on the bacteria, we monitored the flagellar rotation speed of individual cells under different loads captured by the optical tweezer within two intervals: 0–5 s and 5–10 s (the corresponding numbers of bacteria were 50, 45, and 47 for the wild-type strain in 2% Ficoll, Δ*motCD* mutant in 7% Ficoll, and Δ*motAB* mutant in 15% Ficoll, respectively, and the typical examples are shown in Fig. S1 to S3). We observed no obvious difference between the two intervals for the cells in this study. To quantify the impact of the optical trap on the flagellar rotation speed, we calculated the change in the rotation speed of the latter interval relative to the former (Fig. S4). All changes were minimal, with mean relative changes of 1.8%, 1.9%, and 1.9% for the wild-type strain in 2% Ficoll, Δ*motCD* mutant in 7% Ficoll, and Δ*motAB* mutant in 15% Ficoll, respectively. Therefore, we concluded that the laser-induced photodamage on the cell from the optical tweezer was negligible. In addition, we obtained only 1–2 measurements per observed sample after sealing the periphery of the sample with vacuum grease, to minimize the potential negative impact of oxygen depletion on flagellar motor rotation speed.

We determined the output torque of the flagellar rotary motor using the flagellum rotation speed $\omega_{f}$. For swimming at low Reynolds numbers, the motor torque *T* is balanced by the resistive torque *T_f_* from flagellar rotation, calculated as (32, 33) $T=T_{f}=f_{f}\;\times \omega_{f},$ where $f_{f}$ represents the rotational drag coefficient of the filament. This coefficient is determined by the geometrical morphology of the flagellar filament and the viscosity of the medium for the swimming bacterium (refer to Materials and Methods for a detailed calculation of $f_{f}$). We measured the waveform parameters of the flagellar filament, such as the helix radius, contour length, and pitch in various liquid environments (more than 20 flagellar filaments were measured under each load). We found that these parameters were not affected by the viscosity of the medium, as shown in Fig. S5. Further details of the measurements are presented in Materials and Methods. In addition, we defined the rotational drag coefficient of the flagellar motor $f_{motor,t}$ as the ratio of motor torque *T* to the motor rotation speed $\omega_{motor}$. This coefficient represents the external load that the flagellar motor experiences when the cell is trapped by optical tweezers (the calculation of $f_{motor,t}$ is presented in the Materials and Methods section, and the values are presented in Table S1).

To verify the accuracy of our new torque-measurement method in this study, we measured the flagellar motor torque of *Salmonella* strain TH14551. More than 70% of cells in this strain have a single polar flagellum. We used optical tweezers to trap swimming bacterial cells under high load conditions (in MB mixed with 9% Ficoll, where the rotational drag coefficient of the flagellar motor was 17.74 pN⋅nm⋅s). Our calculations showed that the flagellar motor output torque was approximately 1,100 pN⋅nm (see Materials and Methods). This result is consistent with previous measurements for *Salmonella* using the bead assay (34), supporting the reliability of our new method.

### Torque-speed relationship for flagellar motors with dual-stator, MotAB-stator, and MotCD-stator systems

**Fig 2:**
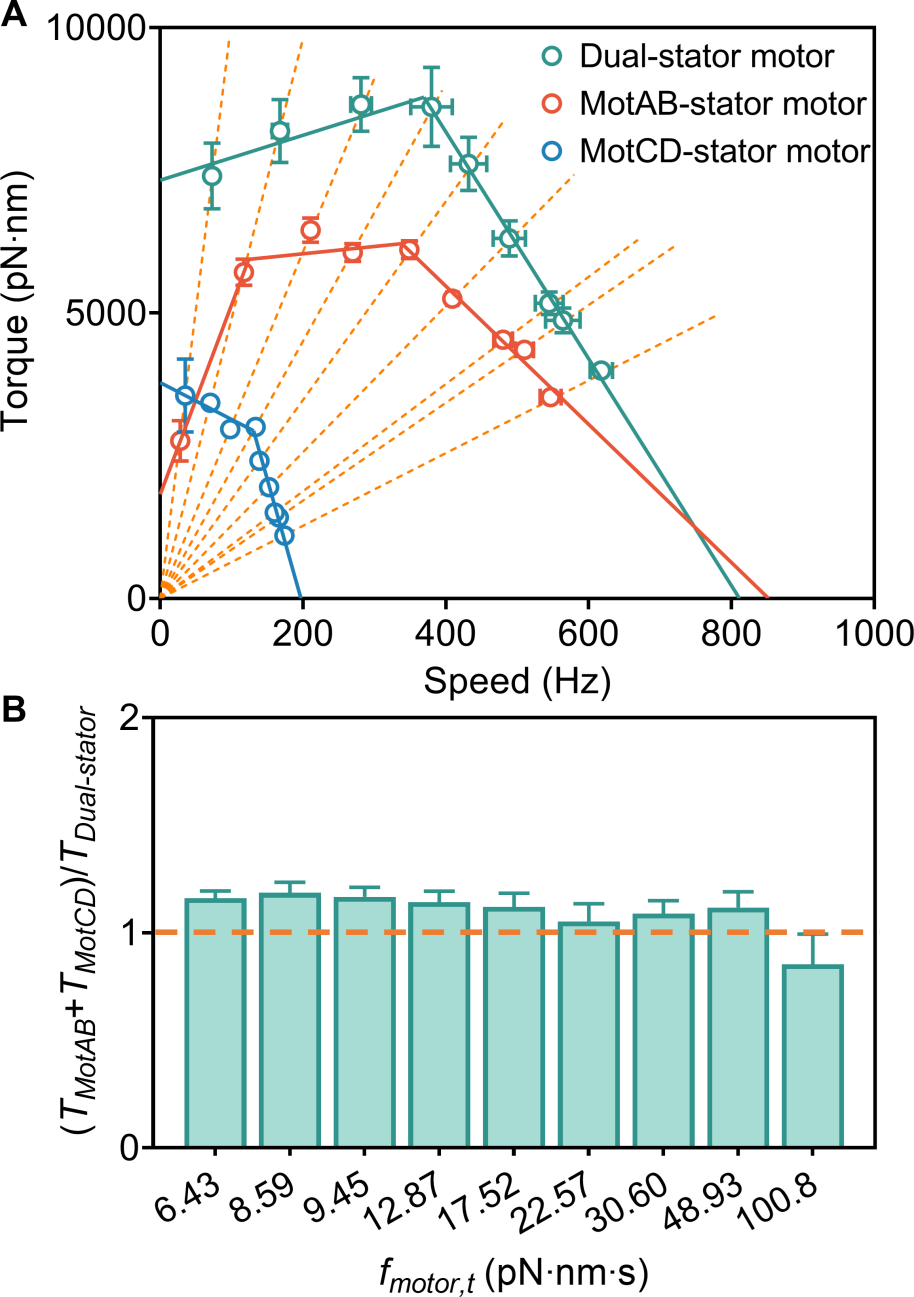
(**A**) The torque-speed relationship of the MotAB-stator (Δ*motCD* mutant, red circles with error bars), MotCD-stator (Δ*motAB* mutant, blue circles with error bars), and dual-stator motor (wild-type strain, cyan circles with error bars). The symbols and error bars represent means and SEMs for the cell population under each load. The solid lines are linear regressions. Each dotted line represents the measurements under a specific load (the same motor rotational drag coefficients $f_{motor,t}$). Each group of data points of the torque-speed curves from right to left represents the measurement results in MB mixed with 0%, 2%, 3%, 5%, 7%, 9%, 12%, 15%, and 20% (wt/vol) Ficoll, respectively, and the corresponding values of the torque and the rotation speed are presented in Table S2. The slopes of the dotted lines represent the motor rotational drag coefficients $f_{motor,t}$. (B) The ratio of the sum of output torque supplied by the MotAB-stator and MotCD-stator mutant motor to the output torque of the dual-stator wild-type motor under different loads. The bars from left to right represent the ratios in MB mixed with 0%, 2%, 3%, 5%, 7%, 9%, 12%, 15%, and 20% (wt/vol) Ficoll, respectively.

We varied the rotational drag coefficients $f_{f}$ of the filament by changing the viscosity of the liquid using Ficoll. Specifically, we measured the $f_{f}$ value for nine different concentrations of Ficoll (wt/vol 0%, 2%, 3%, 5%, 7%, 9%, 12%, 15%, 20%), with the corresponding values presented in Table S1. We measured the torque-speed relationships for the flagellar motor of the wild-type, Δ*motCD*, and Δ*motAB* mutant strains, and the results are plotted in Fig. 2A (values and corresponding numbers of motors are shown in Table S2). The cyan, red, and blue symbols represent the measurements of the motors with dual-stator, MotAB-stator, and MotCD-stator systems, respectively. The slopes of the orange dotted lines represent the rotational drag coefficients $f_{motor,t}$ for motors of bacterium cells trapped by the optical tweezer under different loads. The circles indicate means and SEMs, while the lines represent linear regressions. Interestingly, the three torque-speed curves are drastically different.

The torque-speed relationship of the wild-type motor with dual-stator systems is similar to that of the *E. coli* motor (26, 35). The motor torque remains nearly constant (albeit increasing slightly with speed) between stall and the knee speed of about 370 Hz, before dropping rapidly to 0 at about 810 Hz. This is indicative of a power-stroke mechanism rather than a thermal-ratchet mechanism (23, 36). The stall torque is approximately 7300 pN·nm, more than triple the value of 2,200 pN·nm in *E. coli* (37). The zero-torque speed is 800 Hz, approximately triple that of *E. coli* (35).

The shape of the torque-speed relationship of the flagellar motor with the MotCD stator system is similar to that of the wild-type motor and closely resembles that of the *E. coli* motor. There are also two dynamic regimes: the motor torque drops approximately 24% (from 3800 pN·nm down to 2,900 pN·nm) between stall and the knee speed of about 130 Hz, and then rapidly declines, dropping to 0 at 200 Hz (zero-torque speed). The magnitude of the motor torque with the MotCD stator system differs significantly from that of the wild type but is similar to that of the flagellar motor in *E. coli*.

The shape of the torque-speed relationship of the flagellar motor with the MotAB stator system differs significantly from that of the wild-type motor and the motor with the MotCD stator system. The motor torque increases rapidly from 1,800 pN·nm to 6,000 pN·nm at 120 Hz, remains approximately constant between 120 and 340 Hz, and then swiftly drops to 0 at 850 Hz. The motor torque for the MotAB stator system is notably below that for the MotCD stator system under extremely high load conditions near stall, but it is significantly higher at lower load. This indicated that the MotAB and MotCD stators primarily contribute to providing the motor torque under low load conditions such as swimming in the water and under high load conditions such as swarming on the agar surface, respectively, in agreement with previous studies (19–22).

As the zero-load speed of the MotCD-stator motor is much smaller than that of the MotAB-stator or wild-type dual-stator motors, we were intrigued by the question of how both sets of stators contribute to the output torque in the wild-type cells under different loads, especially when the motor speed exceeds the zero-load speed of the MotCD-stator motor. To investigate this, we compared the output torque of the motors with dual-stator, MotAB-stator, and MotCD-stator systems under different loads (Fig. 2B). The results revealed that the sum of output torque supplied by the Δ*motCD* and Δ*motAB* mutant strains and the output torque of the wild-type strain were comparable under the same load (the same motor rotational drag coefficient $f_{motor,t}$). The ratios of ${\text{T}}_{\text{M}\text{otAB}}\text{+}{\text{T}}_{\text{MotCD}}$ to ${\text{T}}_{\text{Dual-stator}}$ were as follows: 1.16 ± 0.03, 1.19 ± 0.05, 1.17 ± 0.04, 1.14 ± 0.05, 1.12 ± 0.06, 1.05 ± 0.08, 1.09 ± 0.06, 1.12 ± 0.07, and 0.85 ± 0.14 (mean ± SEM) in MB mixed with 0%, 2%, 3%, 5%, 7%, 9%, 12%, 15%, and 20% Ficoll, respectively. The corresponding medium viscosities are shown in Table S1. The results in 0% Ficoll are consistent with previous observations comparing the swimming speeds of the wild-type, Δ*motCD*, and Δ*motAB* strain in water, where the Δ*motCD* strain was found to be swimming slightly slower than the wild type, and the Δ*motAB* strain swam considerably slower than the wild type (20). These findings are surprising. They suggest that the MotCD stators in the wild-type motor do not slow down the motor, even though the motor speed is beyond the zero-load speed of the MotCD stator system. Consider the wild-type motor under a specific external load (indicated by a dashed line in Fig. 2A): the motor torque is similar to the combined torque of MotAB-only and MotCD-only motors under the same load. It seems that each set of stators functions independently according to the external load, regardless of whether the other set of stators is present and affecting the motor speed. These results imply that the two sets of stators are probably recruited independently by the flagellar motor, and there is no interference between the functions of the MotAB and MotCD stators in wild-type *P. aeruginosa* motor. A previous study in *E. coli* reported on the dynamics of a hybrid-fuel motor with dual stator systems (Na^+^-driven MotA/MotB and H^+^-driven PomA/PotB). In that case, the two types of stator units compete for spaces in the motor (38).

To verify the above hypothesis, we upregulated *motC* and *motD* gene expression in the Δ*motAB* mutant strain using a plasmid that expresses MotCD (see Materials and Methods). We found no obvious increase in motor output torque for this strain with overexpressed MotCD compared to wild-type expression under high load conditions. Specifically, the output torque ratios of the Δ*motAB* strain MT20 with overexpressed MotCD to wild-type expression were close to 1 in MB mixed 20%, 15%, 12%, and 9% Ficoll (Fig. S6). This result suggests that overexpression of the *motC* and *motD* genes does not increase MotCD-stator site occupancy, even when the sites that typically bind MotAB stators are unoccupied.

### Slip-bond behavior in the load dependence of the MotAB stator system

An intriguing finding was that the output torque of the motor with MotAB stator system in 20% Ficoll was 2760 pN·nm, significantly below the value of the low-speed plateau of the torque-speed curve (Fig. 2A). This may have resulted from a decrease in the number of stator units binding to the flagellar motor under extremely high loads. To test this hypothesis, we waited for a swimming cell in MB with 7% Ficoll to settle onto the glass and tether *via* its flagellar filament, which abruptly increased the load. The corresponding motor rotational drag coefficient increased from 18 to 700 pN⋅nm.s, switching the load condition from knee load to extremely high load. The calculation of the motor rotational drag coefficients ${\text{f}}_{\text{TC}}$ for tethered cells is presented in Materials and Methods.

**Fig 3:**
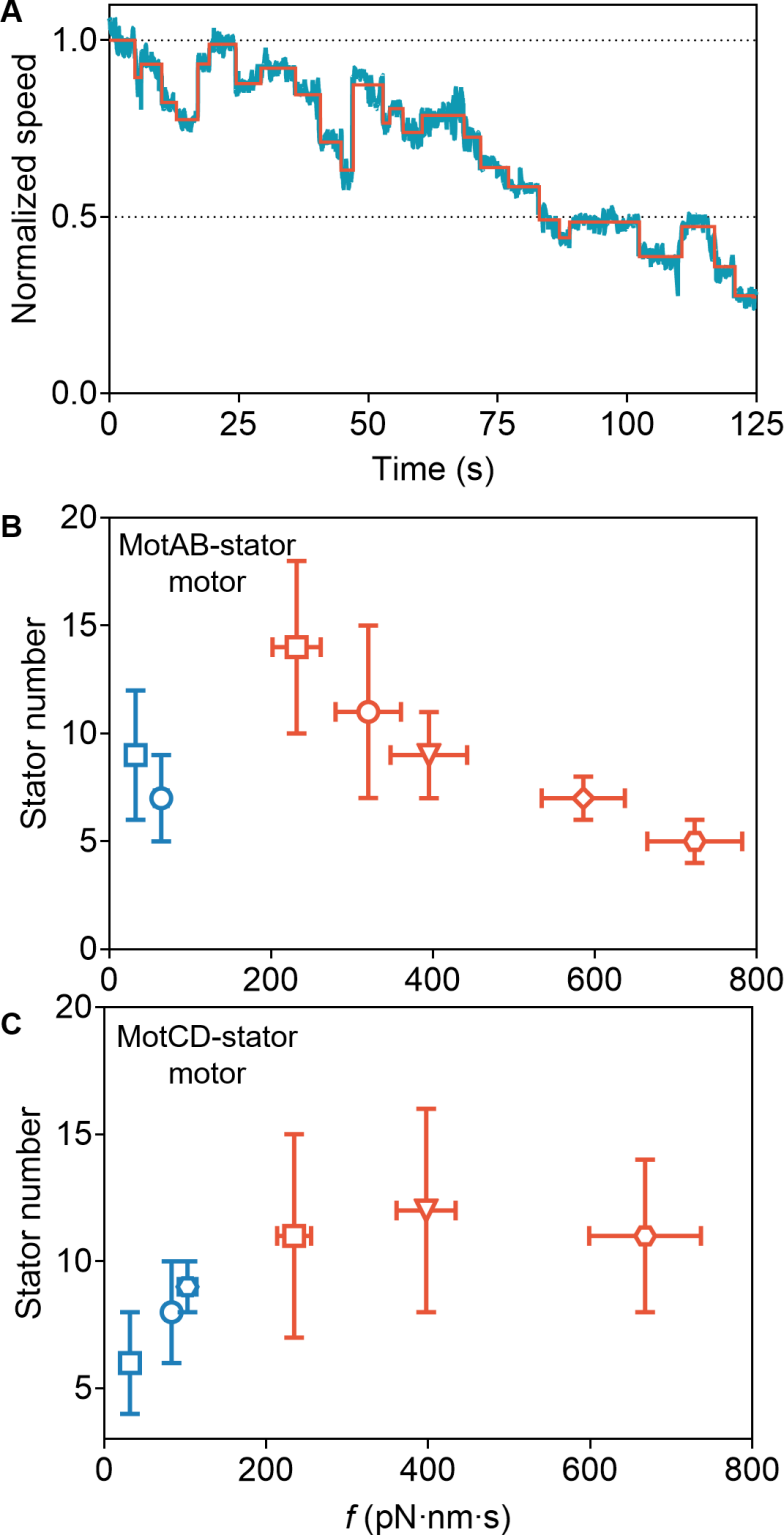
(**A**) A typical rotation trace of a tethered Δ*motCD* mutant cell driven by the flagellar motor when the bacterium had just switched from free swimming to tethering mode in MB with 7% Ficoll. The rotation speed of the cell body was normalized to the average speed of the initial 5 s. The red solid line represents the average speeds found by the step-finding algorithm. (**B**) The steady-state stator number for MotAB-stator motors under various loads. The data left to right are from bead assay in 0% (blue square) and 2.5% Ficoll (blue circle), and tethered-cell assay in 0% (red square), 2% (red circle), 3% (red inverted-triangle), 5% (red rhombus), and 7% Ficoll (red octagon). Error bars of the horizontal axis data represent SEM. The stator numbers are in order as follows: 9 ± 3, 7 ± 2, 14 ± 4, 11 ± 4, 9 ± 2, 7 ± 1, and 5 ± 1 (mean ± std). Data for the bead assay in 0% and 2.5% Ficoll are from reference (21). (**C**). The steady-state stator number for MotCD-stator motors under various loads. The data left to right are from bead assay in 0% (blue square), 5% (blue circle), and 9% Ficoll (blue octagon), and tethered-cell assay in 0% (red square), 3% (red inverted-triangle), and 7% Ficoll (red octagon). Error bars of the horizontal axis data represent SEM. The stator numbers are in order as follows: 6 ± 2, 8 ± 2, 9 ± 1, 11 ± 4, 12 ± 4, and 11 ± 3 (mean ± std). Data for the bead assay in 0% and 5% Ficoll are from Ref. 21.

We tracked the rotation of the tethered cell body driven by the flagellar motor, observing more than 20 bacteria. A typical trace is shown in Fig. 3A, along with the average speeds (red solid lines) determined by the step-finding algorithm described previously (21). We observed a stepwise decline in the rotation speed of the tethered cell body, indicating that the stator unbinding rate increased upon tethering of the cell body, and more stators detached from the motor. This suggested a slip-bond behavior in the *P. aeruginosa* MotAB stator binding to the peptidoglycan, contrasting with the catch-bond behavior in *E. coli* MotAB stator binding (21, 29).

Consistent with this, we measured the steady-state number of stator units in the Δ*motCD* and Δ*motAB* mutant strains under different loads. Specifically, we monitored the rotation traces of tethered Δ*motCD* strain cells at a steady state in MB mixed with 0%, 2%, 3%, 5%, and 7% Ficoll in this study (the typical traces were shown in Fig. S7). We found that stochastic speed jumps were presented, showing a series of speed segments. The mean number of stator units for a motor was determined by dividing the mean speed by the mean speed change per stator for a whole trace. In addition, we included results of the steady-state stator numbers under two different loads using a bead assay from our previous study with a 1-μm-diameter bead in 0%, 2.5%, and 5% Ficoll (21). We also performed additional measurements for the Δ*motAB* strain at steady state with the bead assay using a 1-μm-diameter bead in 9% Ficoll, and tethered-cell assay in MB mixed with 0%, 3%, and 7% Ficoll (the typical traces are shown in Fig. S8). Details of calculating the motor rotational drag coefficient ${\text{f}}_{\text{BA}}$ for the bead assay are presented in Materials and Methods. Taking all these results into account, we found the steady-state numbers of MotCD stators were 6 ± 2, 8 ± 2, 9 ± 1, 11 ± 4, 12 ± 4, and 11 ± 3 (mean ± std) under motor rotational drag coefficient of 31.8, 83.2, 102.9 ± 10.9, 234.4 ± 21.0, 397.4 ± 36.4, and 667.7 pN⋅nm⋅s (mean ± SEM) for the bead assay with 1-μm-diameter beads in MB mixed with 0%, 5%, and 9% Ficoll, and tethered-cell assay in 0%, 3%, and 7% Ficoll, respectively (Fig. 3C). The positive correlation between the stator number and load suggested catch-bond behavior of MotCD stator binding. Conversely, the steady-state numbers of MotAB stators were 9 ± 3, 7 ± 2, 14 ± 4, 11 ± 4, 9 ± 2, 7 ± 1, and 5 ± 1 (mean ± std) under motor rotational drag coefficient of 32.6, 64.2, 231.5 ± 29.8, 320.1 ± 40.3, 394.9 ± 47.3, 585.7 ± 51.4, and 723.4 pN⋅nm⋅s (mean SEM), for the bead assay with 1-μm-diameter beads in MB mixed with 0% and 2.5% Ficoll, and tethered-cell assay in 0%, 2%, 3%, 5%, and 7% Ficoll, respectively (Fig. 3B). The steady-state number of MotAB stators decreases as the load increases at high loads, indicating slip-bond behavior at these high loads. There appears to be a potential increase in the stator number at lower loads, suggesting that the MotAB stators might exhibit catch-bond behavior at these lower loads, although more data at lower loads are needed to firmly establish this.

### Solvent-isotope effects on the torque-speed relationship

The rotation of the flagellar motor is driven by the ion flow across the cytoplasmic membrane (6, 7), which is influenced by both the transmembrane electrical potential and the transmembrane proton concentration gradient. To examine the dependence of bacterial flagellar motor output on the ion flow, we studied the solvent-isotope effects on the motor torque-speed relationship. Previous studies indicated that the solvent-isotope effects were small in the low-speed regime but large in the high-speed regime in flagellar motors of *Streptococcus*, *E. coli*, and *Salmonella typhimurium* (39–43).

**Fig 4:**
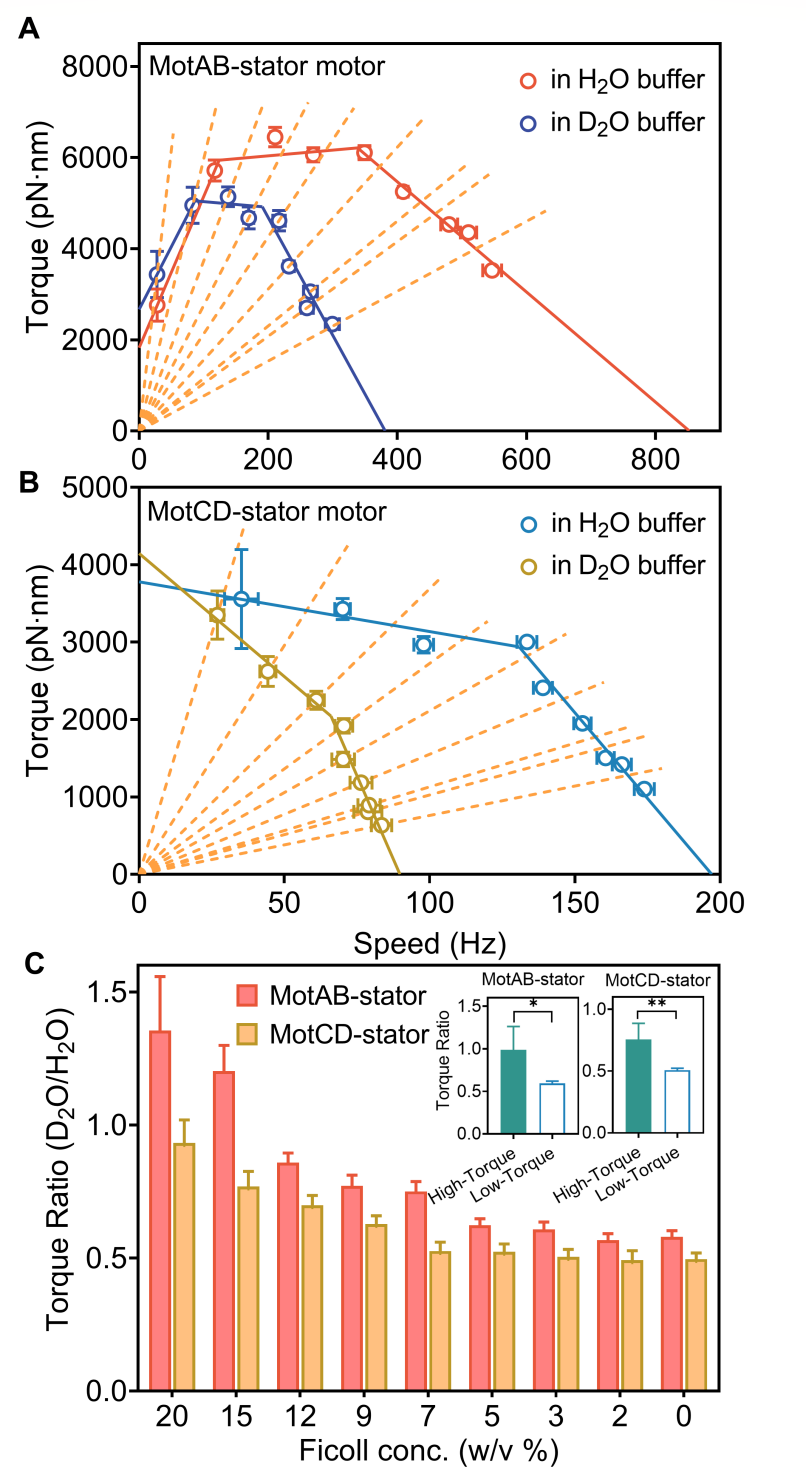
The torque-speed relationship for the MotAB-stator (**A**) and MotCD-stator motors (**B**) in H_2_O and D_2_O media. The symbols and error bars represent means and SEMs for the cell population under each load. The solid lines are linear regressions. Each dotted line represents the measurement under a specific load in the D_2_O medium. (**C**) The ratios of the output torque in D_2_O to that in H_2_O under different loads. The inset shows the mean ratios for MotAB-stator and MotCD-stator motors in the high-torque (speed below the keen speed) regime and low-torque (speed above the keen speed) regime. *P < 0.05; **P < 0.01; ***P < 0.001.

In this study, we replaced H_2_O with the D_2_O medium to measure the torque-speed relationships for motors with either MotAB or MotCD stator systems. To compare with the results in the H_2_O medium at pH 7.0, measurements were performed in the D_2_O medium at pH 7.5. This adjustment compensated for the upward shift of internal pH due to the higher dissociation constants of weak acids and bases in D_2_O compared to H_2_O (40). The measurement results (Fig. 4A and B, values and corresponding numbers of motors are shown in Table S3) indicated similar solvent-isotope effects on the torque-speed curves of both the MotAB and MotCD stator systems. Specifically, the stall torques in the D_2_O medium were nearly identical to those in the H_2_O medium. However, the knee speeds (~200 and 65 Hz for MotAB and MotCD stators, respectively) and the zero-torque speeds (~380 and 90 Hz for MotAB and MotCD stators, respectively) were significantly lower in D_2_O than in H_2_O.

We calculated the ratio of motor torque in the D_2_O medium and H_2_O medium at the same medium viscosities. Because the viscosity ratio at identical Ficoll concentrations in D_2_O and H_2_O is 1.22 at room temperature (40), the output torque in the H_2_O medium was taken from the fitting results of the torque-speed curve under the same viscosities as the D_2_O medium. The results, shown in Fig. 4C, revealed a decrease in the torque ratio with a decreasing load. Furthermore, we calculated the mean ratios of the output torque in two isotope mediums under both high-torque (low-speed) and low-torque (high-speed) regimes (Fig. 4C inset). We discovered significant differences between the mean ratios in these two regimes: 0.99±0.27 and 0.59±0.03 for the MotAB stator (mean std), with a significant difference of *P* < 0.05; 0.76±0.13 and 0.51±0.02 for the MotCD stator (mean std), with a significant difference of *P* < 0.01. This indicated that the solvent isotope effects were small at speeds below the knee speed but large at speeds above the knee speed. It suggested that the decline in torque at high speeds in the torque-speed relationship of the two types of stator systems was primarily due to limitations in the rates of proton transfer. This behavior is akin to that of *E. coli* motors.

### pH effects on the flagellar rotation

**Fig 5:**
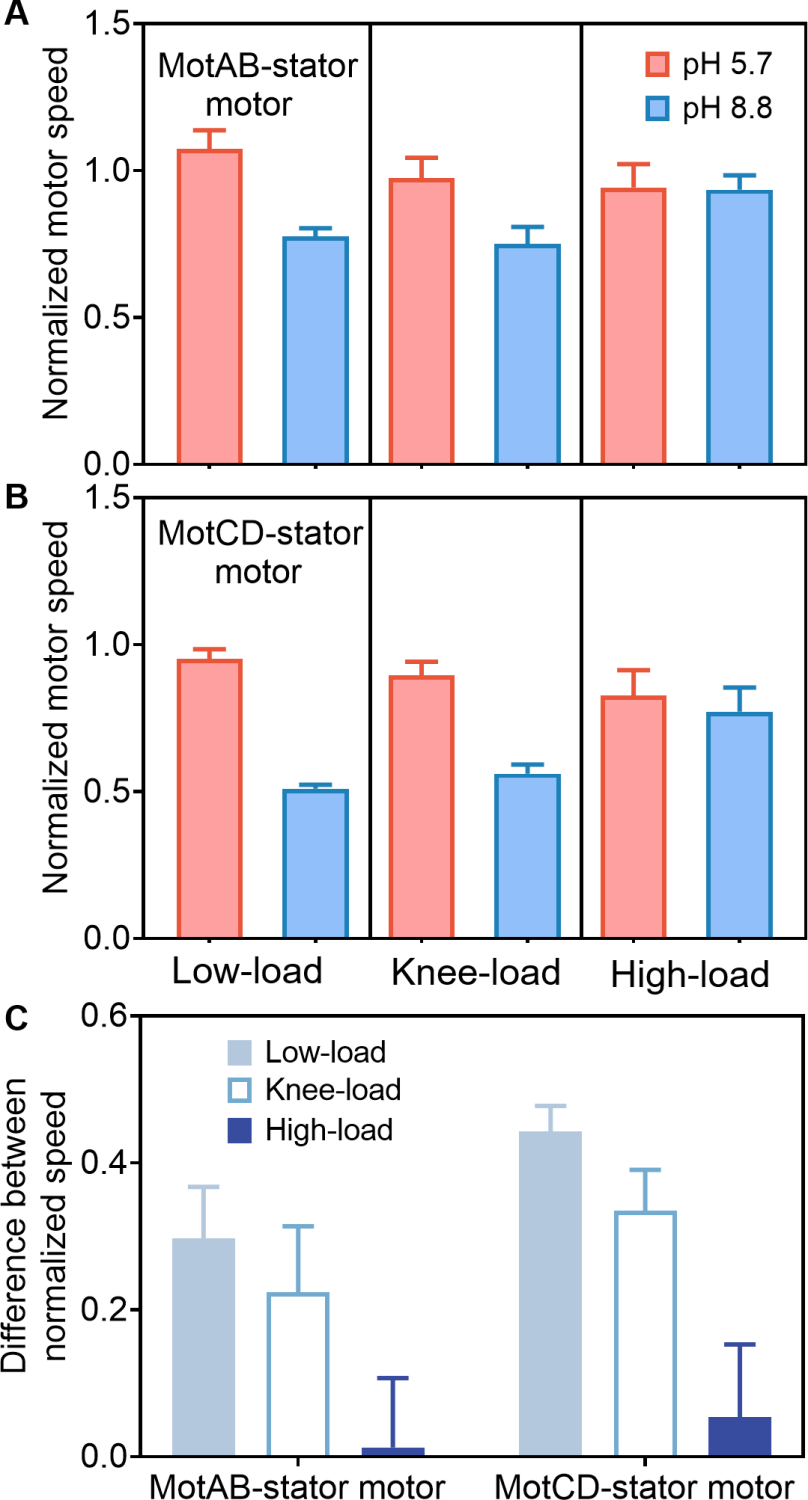
The normalized rotation speed for flagellar motors with MotAB (**A**) and MotCD (**B**) stator systems at different pH values relative to the mean speeds at pH 7.0 under low-load (2% Ficoll), keen-load (7% for MotAB-stator motor and 9% Ficoll for MotCD-stator motor), and high-load (12% for MotAB-stator motor and 15% Ficoll for MotCD-stator motor) conditions. The columns with error bars represent the means and SEMs. (**C**) The difference between normalized rotation speeds at pH 5.7 and 8.8 for flagellar motors with MotAB and MotCD stator systems under low-load, knee-load, and high-load conditions.

We studied the effects of external pH on flagellar rotation for motors with either MotAB or MotCD stator systems. We measured the rotation speeds of these two types of stator systems under different loads at pH levels of 5.7, 7.0, and 8.8. Measurements for low load, knee load, and high load were performed for the high-speed (2% Ficoll for both types of stator systems), keen-speed (7% Ficoll for MotAB-stator motor and 9% for MotCD-stator motor), and low-speed regimes (12% Ficoll for MotAB-stator motor and 15% for MotCD-stator motor), respectively. We normalized the results at other pH levels to the mean speed value at pH 7.0 under the same load. The results (Fig. 5, with values and corresponding numbers of motors shown in Table S4) revealed that as pH increased from 5.7 to 8.8, the speed decreased more for motors under low loads than those under high loads. The difference in normalized speed at pH 5.7 and 8.8 was 0.297 for the MotAB-stator motor and 0.443 for the MotCD-stator motor under low loads, but only 0.012 for the former and 0.054 for the latter under high loads. Both stator systems in the high-load (low-speed) regime were less affected by changes in external pH than those in the low-load (high-speed) regime. This behavior is similar to that in *E. coli* (40, 44, 45).

Bacteria maintain homeostasis of both internal pH and the proton motive force. As the external pH increases from 5.7 to 8.8, the contribution to the proton motive force from the transmembrane pH gradient decreases, while the transmembrane electrical potential increases (40). The pH effects we observed here suggest the non-equivalence of the transmembrane pH gradient and electrical potential for motors at low loads, indicating that proton transfer may be the rate-limiting step at low loads. This is consistent with the solvent-isotope effects we observed above and aligns with the non-equivalence of the two components of the proton motive force found in *E. coli* motors (44). In addition, titratable groups near the mouth of the ion channel on the extracellular side could play a role, as their protonation states may modulate the openness of the channel and thus affect the rate of the motor’s turnover cycle.

## SUMMARY AND DISCUSSION

In this work, we measured the torque-speed relationships for wild-type motors with dual-stator systems and motors with the MotAB or MotCD stator system in *P. aeruginosa*. We accomplished this by trapping the swimming bacterium using an optical tweezer and by fluorescently labeling the flagellar filament. We found that their torque-speed relationships are drastically different. The torque-speed relationship for the MotCD-stator motors is similar to that of the *E. coli* motor with MotAB stators, exhibiting two dynamic regimes. The motor torque drops approximately 24% (from 3,800 pN·nm down to 2,900 pN·nm) between stall and the knee speed of about 130 Hz, and then rapidly declines, dropping to 0 at 200 Hz (zero-torque speed). By contrast, the torque-speed relationship for the MotAB-stator motors differs significantly from that of the MotCD-stator motors. The motor torque increases rapidly from 1,800 pN·nm to 6,000 pN·nm at 120 Hz, remains approximately constant between 120 and 340 Hz, and then swiftly drops to 0 at 850 Hz. Interestingly, the torque for the wild-type motor appears to be close to the sum of the torques provided by each set of stators under a specific load. As such, the wild-type motor torque remains nearly constant (albeit increasing slightly with speed) between stall and the knee speed of about 370 Hz, before dropping rapidly to 0 at about 810 Hz.

The stall torque we observed in *P. aeruginosa* is remarkably high compared to that of *E. coli*, which is surprising and challenging to explain based on current understanding. A previous study found that the size of the *P. aeruginosa* motor C-ring was very similar to that of *E. coli* (46). Given that the pmf in *P. aeruginosa* is unlikely to be three times that of *E. coli*, and the structural constraints limit substantial increases in the stator or FliG numbers, the mechanisms underlying the high stall torque of the *P. aeruginosa* motor need further investigation.

At extremely high loads, the torque for motors with MotAB stators drops rapidly as the load increases, contrasting directly with the behavior of motors with MotCD stators or *E. coli* motors with MotAB stators. This indicates a slip-bond behavior in the load-dependent binding of the *P. aeruginosa* MotAB stators to the motor, in contrast to the catch-bond behavior exhibited by the *P. aeruginosa* MotCD stators and the *E. coli* MotAB stators. This observation seems antithetical to the previous finding that there is a higher sequence similarity between *E. coli* MotAB and *P. aeruginosa* MotAB than MotCD (20). This suggests that the similarity of stator-binding behaviors among different flagellar motors might depend on specific sites associated with the mechano-sensitivity of the stator complex, rather than the homology of the entire stator. We will verify this hypothesis below by aligning the stator sequences (Fig. S9). We confirmed further this slip-bond behavior by observing the stepwise decrease in stator number when the load on *P. aeruginosa* motors with MotAB stators abruptly increased, as the cell suddenly tethered on the glass *via* its flagellar filament. We further corroborated this observation by measuring the steady-state stator number under seven different load conditions for *P. aeruginosa* motors with MotAB stators. Moreover, we found that MotAB stators might exhibit catch-bond behavior at lower loads.

This unusual behavior of the *P. aeruginosa* Δ*motCD* mutant motor was similar to that observed in the *Salmonella* MotA-E155K mutant motor, as reported in a previous study (34). In *Salmonella*, the MotA (E155K) mutation converts the stator phenotype from a catch bond to a slip bond under extremely high load. The MotA-FliG interaction occurs *via* a cytoplasmic loop in MotA, and mutations in this domain are likely to affect the mechanical sensing of the stator complex. To explore this molecular basis, we aligned the sequences of *P. aeruginosa* MotA/MotC proteins with those of *E. coli* MotA and *Salmonella* MotA. This alignment revealed conserved residues corresponding to Salmonella’s MotA-E155, specifically PAO1-MotA-K154, PAO1-MotC-E136, and K12-MotA-E155 (Fig. S9). The strong site conservation between *P. aeruginosa* MotC and *E. coli/Salmonella* MotA explains the catch bond phenotype of the MotCD complex. Interestingly, the presence of Lys154 in *P. aeruginosa* MotA is analogous to the E155K mutation in *Salmonella*, which is known to induce slip-bond behavior under extremely high load. This molecular similarity provides a theoretical framework for understanding how the MotAB complex in *P. aeruginosa* exhibits slip-bond behavior under high loads, similar to the mutated MotAB stators in *Salmonella*.

Such an autonomous stator exchange mechanism observed in the *P. aeruginosa* flagellar motor may be conserved among bacterial species possessing dual stator systems. For instance, the wild-type *B. subtilis* operates a hybrid flagellar motor composed of both H^+^-type MotAB and Na^+^-type MotPS stator units under external Na^+^ concentrations (47). The number of MotPS stator units around the rotor increases with load, suggesting catch-bond behavior, similar to the *P. aeruginosa* MotCD system. Conversely, the number of MotAB stator units decreases with increasing load, indicating slip-bond behavior at extremely high load, similar to the *P. aeruginosa* MotAB system (48). As a result, the wild-type *B. subtilis* motor maintains a constant torque over a wide range of load conditions, reminiscent of the behavior observed in *P. aeruginosa* (49).

This autonomous stator exchange mechanism is further modulated by specific functional proteins of the flagellar motor. For example, FliL is a modulator of flagellar motor function in several bacteria species and interacts directly with stator proteins. Previous studies have demonstrated that FliL plays a unique role in high-viscosity environments. Notably, *fliL* mutant and wild-type cells exhibit viscosity-dependent differences in motility phenotypes (50, 51). Based on these observations, it is speculated that when the external viscosity changes, stator-interacting proteins such as FliL may influence stator system selection and regulate the number of bound stator units.

In the low-load (high-speed) regime, the output torque of motors with the MotAB stators was far greater than that of the MotCD stators. This indicates that MotAB stators play a dominant role at low loads, such as when bacteria are swimming in free-liquid environments with a viscosity close to water. By contrast, the output torque of the MotAB stators declined and was lower than that of the MotCD stators at extremely high loads. This is due to the different load-dependent binding to the motor for the two types of stators, suggesting that MotCD stators play a primary role in providing high output torque under high load conditions, such as swarming on the agar surface. Therefore, the different load dependencies for the two stator systems allow wild-type motors to rapidly adjust their stator composition according to the external load conditions, rather than by a slower process of adjusting gene expression, for example, assembling the lateral flagella as in *Aeromonas hydrophila* and *Vibrio parahaemolyticus* (52–54).

To investigate the main factors influencing the dynamics of the MotAB/MotCD stator units, we examined the deuterium solvent-isotope and pH effects on flagellar motor rotation in *P. aeruginosa*. Specifically, we measured the torque-speed relationship of the motors with MotAB or MotCD stators in the D_2_O medium and compared it to that in the H_2_O medium. We found the D_2_O medium did not affect the overall shape of the torque-speed curve. However, the solvent-isotope effects on the output torque of the two types of stator systems were minimal at high loads and substantial at low loads, significantly lowering the knee speed and zero-load speed. This suggests that proton transfer was the rate-limiting step at low loads. We also studied the pH effects on motor output torque for the two types of stator systems under different loads at pH 5.7, 7.0, and 8.8. We found that the pH effects were more pronounced for the flagellar motor in the low-load regime than in the high-load regime, suggesting a non-equivalent contribution of the transmembrane electrical potential and pH gradient to the proton motive force for the motor at low loads. The solvent-isotope and pH effects on the *P. aeruginosa* motors are similar to those on the *E. coli* motors.

An intriguing phenomenon was observed in the output torque of the wild-type motor compared to that of the motor with MotAB or MotCD stators: under a specific load, the former is close to the sum of the latter. This is particularly surprising under low load conditions when the speed of the wild-type motor exceeds the zero-load speed of the MotCD-stator motor. One might expect that the MotCD stator units would interfere with the motor function by generating negative torque beyond the zero-load speed of the MotCD-stator motor. The physical mechanisms behind this synergistic contribution of the two types of stators in the wild-type *P. aeruginosa* motors warrant future investigation.

The characterization of the MotAB and MotCD stator systems in this study reveals novel mechanisms that enable *P. aeruginosa* to adapt its flagellar motor to the demands of its lifestyle as a ubiquitous environmental bacterium and opportunistic pathogen. As an opportunistic pathogen, *P. aeruginosa* must navigate diverse host environments during the course of an infection. The MotAB stators appear to be optimized for swimming in low-viscosity fluids such as water, while the MotCD stators can maintain torque under high-load conditions, which may be encountered in mucus layers or biofilms. The slip-bond behavior of MotAB contrasts with the catch-bond behavior of MotCD, allowing for rapid adjustments in stator composition in response to changes in external load.

This ability to fine-tune motor output through the dynamic exchange of optimized stator units likely contributes to *P. aeruginosa*’s capacity to inhabit a broad range of niches, from aqueous environments to dense, viscous tissues in host organisms. The torque-speed characteristics of the flagellar motor, especially in high-load conditions such as those encountered in viscous environments within the host (e.g., mucus-covered tissues), along with the synergistic contribution of both stator types to the torque output, are critical for the bacterium’s ability to navigate and establish infections. These characteristics contribute to the effectiveness of *P. aeruginosa* as a pathogen and its ability to thrive in a wide range of environments, both inside and outside of host organisms.

## MATERIALS AND METHODS

### Strains and plasmids

All strains of the *P. aeruginosa* used in this work are derived from the wild-type strain PAO1 (21, 33). An in-frame deletion of *fliC* and *cheY* was generated to produce the strain MT12. Nonpolar deletions of *motAB* and *motCD* in MT12 were generated to yield MT20 and MT21, respectively. The mutant *fliC^T394C^* was cloned into the vector pJN105 (Gm^r^), with expression controlled by the PBAD promoter, resulting in the plasmid pMT5. The wild-type stator gene *motC-motD* was cloned into the vector pUCP20 (Tc^r^), with expression controlled by the T7 promoter and *lac* operator, resulting in the plasmid pMT7. For measuring torque-speed relationships of the wild-type, MotAB, and MotCD stator systems, MT12, MT21, and MT20 carrying pMT5 were used. For the tethered cell assay, MT20 and MT21 carrying pMT5 were used. The plasmid pMT5 was electroporated into MT12, MT20, and MT21 for measuring the geometrical morphology of the flagellar filament by labeling the flagella and fluorescence imaging. Nonpolar deletions of *motCD* in MT20 were generated to yield MT23, which was used for functional verification of the plasmid pMT7. Specifically, pMT7 was electroporated into MT23. The resulting strain recovered motility in liquid, confirming the functionality of the plasmid pMT7. Furthermore, MT23 harboring pMT7, when induced with 200 µM IPTG, exhibited swimming behavior similar to MT20. This suggests that the expression level of *motC-motD* genes from pMT7 under these conditions is comparable to their native expression in the MT20 chromosome. To investigate the independent recruitment mechanism for the two sets of stators in the flagellar motor, pMT7 was electroporated into MT20, and 200 µM IPTG was added to upregulate the expression of *motC-motD* genes. In addition, to verify the accuracy of our torque-measurement method, we used the *Salmonella* strain TH14551 (a gift from Kelly Hughes), in which more than 70% of cells have a single polar flagellum, as confirmed by fluorescent labeling. TH14551 is *Salmonella* (*Δhin-5717::FRT*) carrying the plasmid pLM2434 (a gift from Linda McCarter) that expresses the *Vibrio flhFG* genes under the control of a *pTrc* promoter. Expression of *Vibrio flhF* and *flhG* in *Salmonella* results in strains with a single polar flagellum.

### Cell culture and labeling of flagella

A single-colony isolate was grown in 3 mL of LB broth (1% Bacto tryptone, 0.5% yeast extract, and 1% NaCl) overnight to saturation on a rotary shaker (250 rpm at 37°C). An aliquot was diluted 1:100 into 10 mL of LB broth (with the appropriate 30 µg/mL of gentamicin to avoid plasmid loss and 0.01% (wt/vol) of Arabinose to induce the plasmid pJN105 expression) and grew to exponential phase (OD_600_ between 0.9 and 1.0). Cells were harvested by centrifugation 1, 000 × *g* for 10 min, then washed twice in an equal volume of motility buffer [MB: 50 mM potassium phosphate, 15 µM ethylenediaminetetraacetic acid (EDTA), 0.15 M NaCl, 5 mM Mg^2+^, and 10 mM lactic acid (pH 7.0)] (30, 55), and resuspended in 2 mL of this medium.

Flagellar filaments were labeled by following the protocol described previously (56). 1 mL of cells in exponential phase was washed as described above and resuspended in 100 µL of motility buffer. Alexa Fluor 568 maleimide was added to a final concentration of 20 µg/mL, and labeling was allowed to proceed for 30 min at room temperature, with gyration at 80 rpm. Then, the unused dye was removed by washing the cells with motility buffer three times, and the final pellet was resuspended in motility buffer. The sample chamber for fluorescence imaging was constructed using a coverslip (24 × 24 mm) supported by two strips of 1-mm-thick double sticky tape on a glass slide. The periphery of the chamber was additionally sealed with Apiezon vacuum grease. The chamber was put on a Nikon Ti-E inverted fluorescence microscope with the filter set for fluorescein, a 100× oil-immersion objective, and a sCMOS camera (Primer95B; Photometrics) at 200, 80, 40, and 40 fps for bacterium cell swimming in MB with 0%, 7%, 15%, and 20% Ficoll, respectively.

### The measurement of the geometrical morphology of flagellar filament

To determine whether the geometrical morphology of the flagellar filament was affected by medium viscosity, we measured the filament helix radius, contour length, and pitch of the strain MT12 carrying pMT5 in MB mixed with 0%, 7%, 15%, and 20% Ficoll. An example illustrating the procedure for processing the fluorescence images of filaments with MATLAB (Mathworks) is shown in Fig. S5A through C. We adjusted the image contrast, conducted image binarization, and performed sinusoidal function fitting for the wave profile of the filament as described previously (57). We were able to calculate the filament helix radius, contour length, and pitch using the wavelength, amplitude, and waveform number (Fig. S5D through F). There were no obvious differences in the filament helix radius (0.351 ± 0.028, 0.344 ± 0.029, 0.347 ± 0.019 and 0.356 ± 0.013 µm, mean ± SEM), pitch (1.535 ± 0.069, 1.503 ± 0.074, 1.507 ± 0.042, and 1.519 ±0.045 µm, mean ± SEM), and contour length (6.168 ± 0.161, 6.304 ± 0.144, 6.236 ± 0.089, and 6.376 ± 0.127 µm, mean ± SEM) for bacterium cells in different viscosity buffers (mixed with 0%, 7%, 15%, and 20% Ficoll). This indicated that the flagellar filament was not affected by the liquid viscosity. Therefore, the mean values for filament helix radius (*R*), contour length (*L*), and pitch (*p*) of 0.350, 6.281, and 1.514 µm were used for subsequent calculations.

### The calculation of the rotational drag coefficients

The output torque of the flagellar motor is T=fω, where ω is the rotation speed of the flagellar motor, and *f* represents the external load experienced by the motor. In this study, we calculated the *f* value to compare the loads under different experimental conditions: $f_{motor,t}$ for swimming cells trapped by optical tweezers, $f_{TC}$ for tethered cells, and $f_{BA}$ for motors labeled by 1-μm-diameter beads. For ease of comparison across these conditions, the units for ω and *f* are Hz and pN⋅nm⋅s, respectively.

For the swimming cells trapped by an optical tweezer, the torque $T_{f}$ exerted on the flagellar filament is equal in magnitude to the torque $T_{b}$ exerted on the cell body. Thus, the output torque T generated by the flagellar motor is


$$T=T_{f}=T_{b},$$

$$T_{f}=f_{f}\omega_{f},$$

$$T_{b}=f_{b}\omega_{b},$$

where $f_{f}$ and $f_{b}$ are the rotational drag coefficient of the flagellar filament and cell body, respectively. $f_{f}$ is calculated using (58):


$$f_{f}=\frac{2\pi \eta L}{\left[\ln\left(2p/r\right)-0.5\right]\left(4\pi^{2}R^{2}+p^{2}\right)}\left(4\pi^{2}R^{2}+2p^{2}\right)R^{2},$$

where the *R, p, r,* a, *L,* and η represent the filament helix radius, pitch, cross-sectional radius, contour length of the filament, and the viscosity of the medium, respectively. The *r* value was 5 nm, measured by Atomic Force Microscopy (AFM), as described previously (33). The η values of different concentrations of Ficoll are shown in Table S1. Then, $f_{b}$ can be obtained by the measurement results of the rotation speeds of the cell body $\omega_{b}$ and flagellar filament $\omega_{f}$. In our study, the motor speed $\omega_{motor}$ is the sum of $\omega_{b}$ and $\omega_{f}$. The output torque T generated by the flagellar motor is


$$T=f_{motor,t}\omega_{motor},$$

so the rotational drag coefficient for motors of bacterium cells trapped by an optical tweezer $f_{motor,t}$ is


$$f_{motor,t}=\frac{f_{b}f_{f}}{f_{b}+f_{f}}.$$

The $f_{motor,t}$ values under different loads are shown in Table S1, and are very close to the corresponding $f_{f}$ values as $\omega_{b}\ll \omega_{f}$ and $f_{b}\gg f_{f}$.

The motor rotational drag coefficient $f_{TC}$ of the tethered cell was calculated using (31, 59, 60):


$$f_{TC}=2\pi \times \left(\frac{8\pi \eta a^{3}}{3\left[\ln\left(2a/b\right)-0.5\right]}+\frac{8\pi \eta as^{2}}{\ln\left(2a/b\right)+0.5}\right),$$

where the cell body was modeled as an ellipsoid with major axis 2a (the cell body length was measured in this study) and minor axis 2*b* (the cell body width was 0.8 µm) (33), and *s* represents the displacement between the cell body centroid and the rotation center along the long body axis (measured in this study). In the above formula, the first item is the rotational drag coefficient of the cell body about the short body axis, and the second item represents the translational drag coefficient of the cell body along the direction perpendicular to the long body axis.

The motor rotational drag coefficient $f_{BA}$ for the bead assay was calculated using:


$$f_{BA}=2\pi \times \left(8\pi \eta r_{b}^{3}+6\pi \eta r_{b}r_{r}^{2}\right),$$

where the $r_{b}$ and $r_{r}$ represent the radius of the bead attached to the sheared flagellar stubs ($r_{b}$was 0.5 µm) and the radius of the fitting circle for the rotation trajectory of the bead center (measured in this study), respectively.

### Tethered cell assay

We used the same sample chamber as described above for this assay. The strain MT21 carrying plasmids pMT5 was diluted 1:100 into the motility buffer after harvested, and then infused into the sample chamber. The bacteria swimming near the coverslip surface was observed by a Nikon Ti-E phase-contrast microscope with a 40× objective, and recorded with a sCMOS camera (C11440; Hamamatsu Photonics, Hamamatsu, Japan) at 100 fps. We monitored the dynamic process in which the flagellar filament of the swimming cell adhered to the surface and drove the cell body to rotate in the direction of the surface’s normal vector. Subsequently, we calculated the time sequence of the cell body rotation speed. Data analysis was carried out with custom scripts in MATLAB (Mathworks).

### Torque-speed relationship measurements

The swimming cell was trapped by a commercially available multiple optical tweezers setup (NanoTracker; JPK Instruments, Berlin, Germany) (Fig. 1A) (61, 62). The 1,064 nm fiber laser was focused into a diffraction-limited spot with a 60× N.A. 1.0 water immersion objective in the optical tweezer. The trap signal was recorded by a QPD (InGaAs quadrant photodiodes) for at least 10 s at a sampling frequency of 5,000 Hz (for rotation speed lower than 300 Hz) and 10,000 Hz (for rotation speed more than 300 Hz). We calculated the rotation speed of the flagellar filament using the signal data for the first 5 s. To capture the swimming bacterium and avoid potential photodamage, we selected 250, 450, and 650 mW of laser power for the optical tweezer for the experimental measurements in MB mixed with lower than 10%, 12%–15%, and 20% Ficoll, respectively. In addition, we obtained only 1–2 measurements per observed sample after sealing the periphery of the sample with vacuum grease, minimizing the potential impact of oxygen depletion on the flagellar motor rotation speed.

### Verification of the torque measurement method

To verify the accuracy of our new torque measurement method, we measured the flagellar motor torque of *Salmonella* strain TH14551. We labeled the flagellar filament and cell body using Alexa Fluor 488 NHS Ester, following the staining protocol described previously (56). We found that more than 70% of cells have a single polar flagellum, while the remaining cells have 2–3 flagella. We measured the geometrical morphology of the flagellar filament for 20 bacterial cells (an example is shown in Fig. S10A and B). The filament helix radius, contour length, and pitch were 0.255 ± 0.007, 7.228 ± 0.387, and 2.401 ± 0.072 µm (mean ± SEM), respectively. The cross-sectional radius of the filament for *Salmonella* was 6.5 nm, as reported in a previous study (63).

Using our optical trap method, we measured the flagellar rotation speeds of about 200 swimming bacteria in MB mixed 9% Ficoll. The distribution of rotation speed was fitted with a multimodal Gaussian curve (Fig. S10C). The lower peak (61 Hz) was approximately two-thirds of the higher peak (98 Hz). We consider the former and latter to represent the average rotation speeds of single flagella and flagellar bundles, respectively, which is consistent with previous findings for *E. coli* (31). Our calculations showed that the flagellar motor output torque was approximately 1,100 pN.nm.

## SUPPLEMENTAL MATERIAL

Supplemental figures and tables mbio.00745-24-s0001.docx Figures S1-S10 and Tables S1-S4.

ASM does not own the copyrights to Supplemental Material that may be linked to, or accessed through, an article. The authors have granted ASM a non-exclusive, world-wide license to publish the Supplemental Material files. Please contact the corresponding author directly for reuse.
