# Supplementary material for: Torque-speed relationship of the flagellar motor with dual-stator systems in Pseudomonas aeruginosa
Source: mBio. 2024 Oct 30;15(12):e00745-24. doi: 10.1128/mbio.00745-24 (PMC11633141; doi:10.1128/mbio.00745-24)
Supplement: Supplemental figures and tables — Figures S1-S10 and Tables S1-S4. [file mbio.00745-24-s0001.docx]

**Supporting Information**


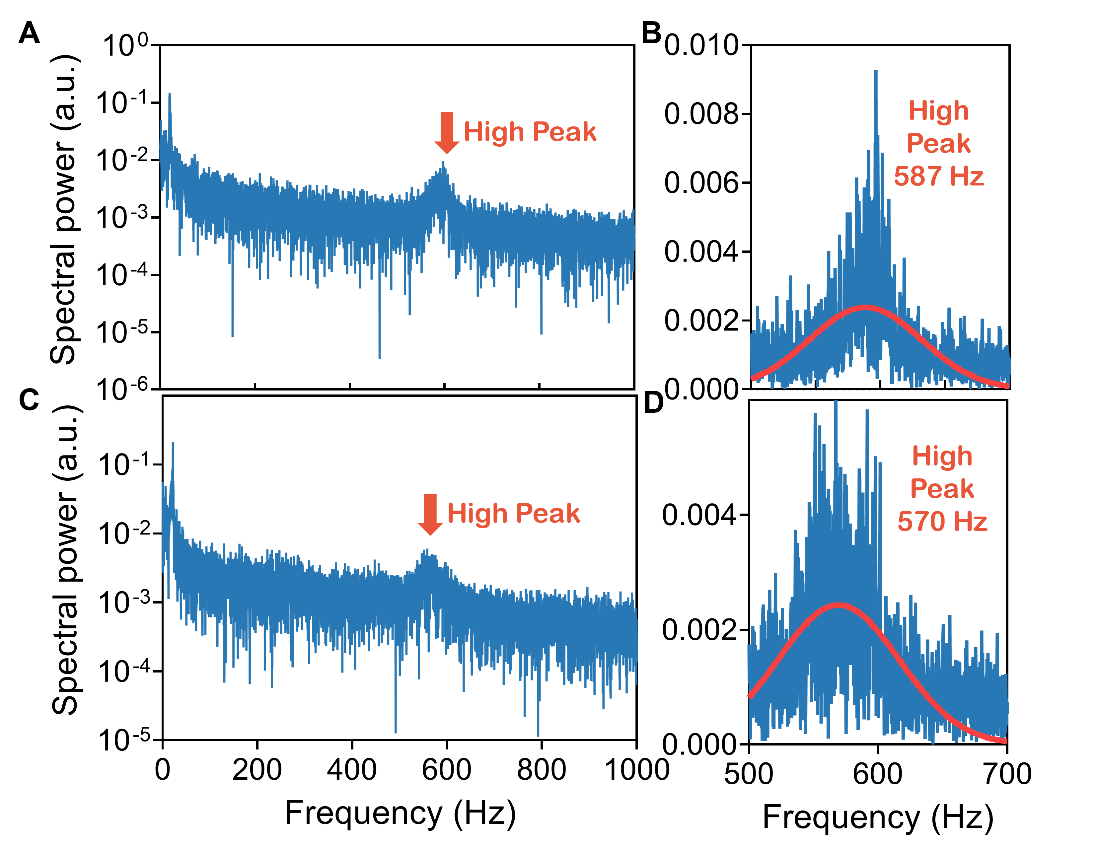


**Fig. S1. A.** and **C.** Typical optical trap signals for a wild-tpye cell along the *x*-direction under low loads (in MB mixed with 2% Ficoll), captured by the optical tweezer within 0 to 5 s and 5 to 10s, respectively. **B.** and **D.** Zoom-in view of the high peak in (A) and (C), respectively. The red solid lines represent Gaussian fits.


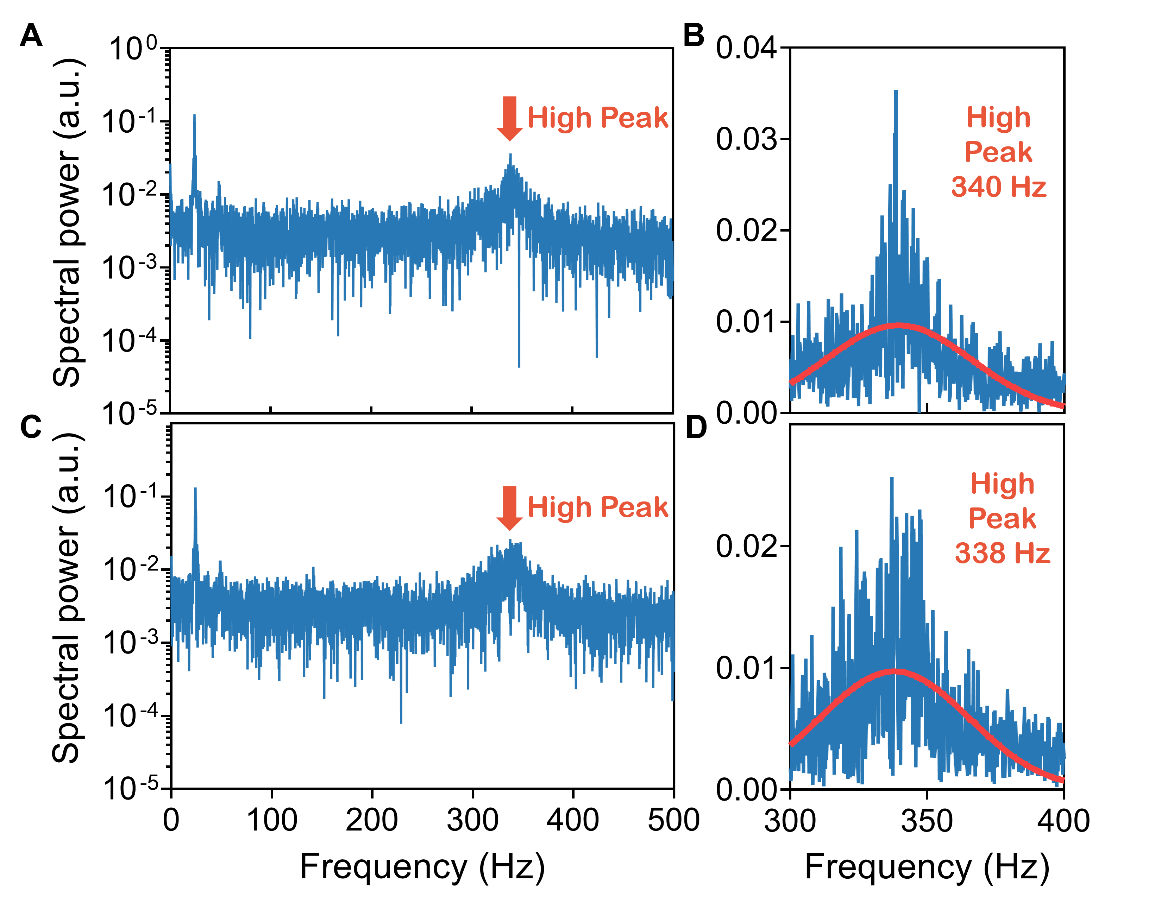
**Fig. S2. A.** and **C.** Typical optical trap signals for a Δ*motCD* mutant cell along the *x*-direction under knee loads (in MB mixed with 7% Ficoll), captured by the optical tweezer within 0 to 5 s and 5 to 10s, respectively. **B.** and **D.** Zoom-in view of the high peak in (A) and (C), respectively. The red solid lines represent Gaussian fits.


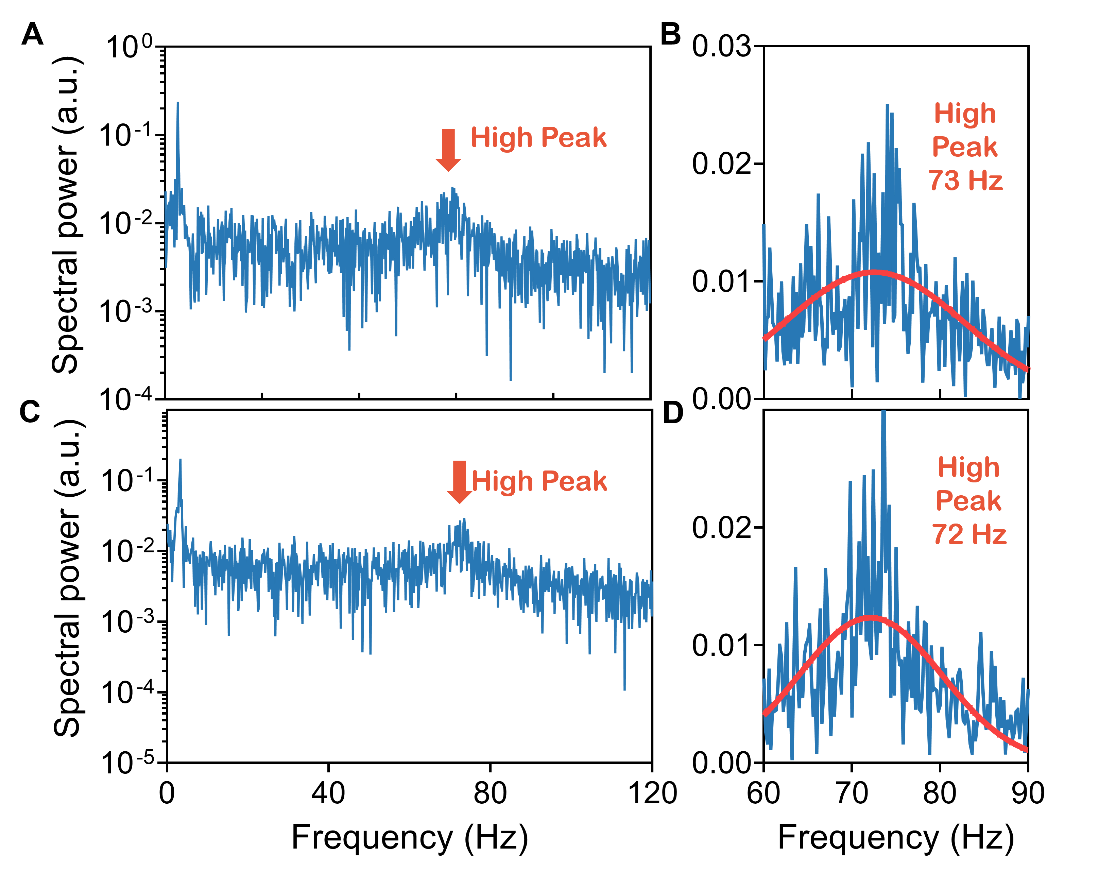
**Fig. S3.** **A.** and **C.** Typical optical trap signals for a Δ*motAB* mutant cell along the *x*-direction under high loads (in MB mixed with 15% Ficoll), captured by the optical tweezer within 0 to 5 s and 5 to 10s, respectively. **B.** and **D.** Zoom-in view of the high peak in (A) and (C), respectively. The red solid lines represent Gaussian fits.


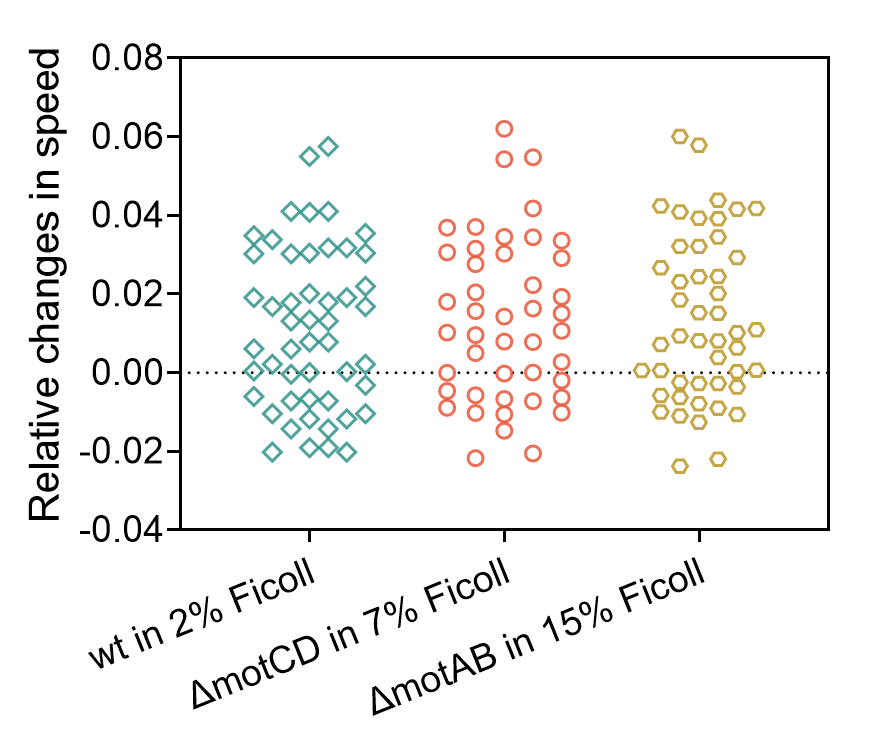
**Fig. S4.** The changes in flagellar rotation speed captured by the optical tweezer within 5-10 s relative to that within the first 5 s, for wild-type strains in 2% Ficoll, *ΔmotCD* mutants in 7% Ficoll, and *ΔmotAB* mutants in 15% Ficoll, and the corresponding numbers of motors were 50, 45, and 47, respectively.


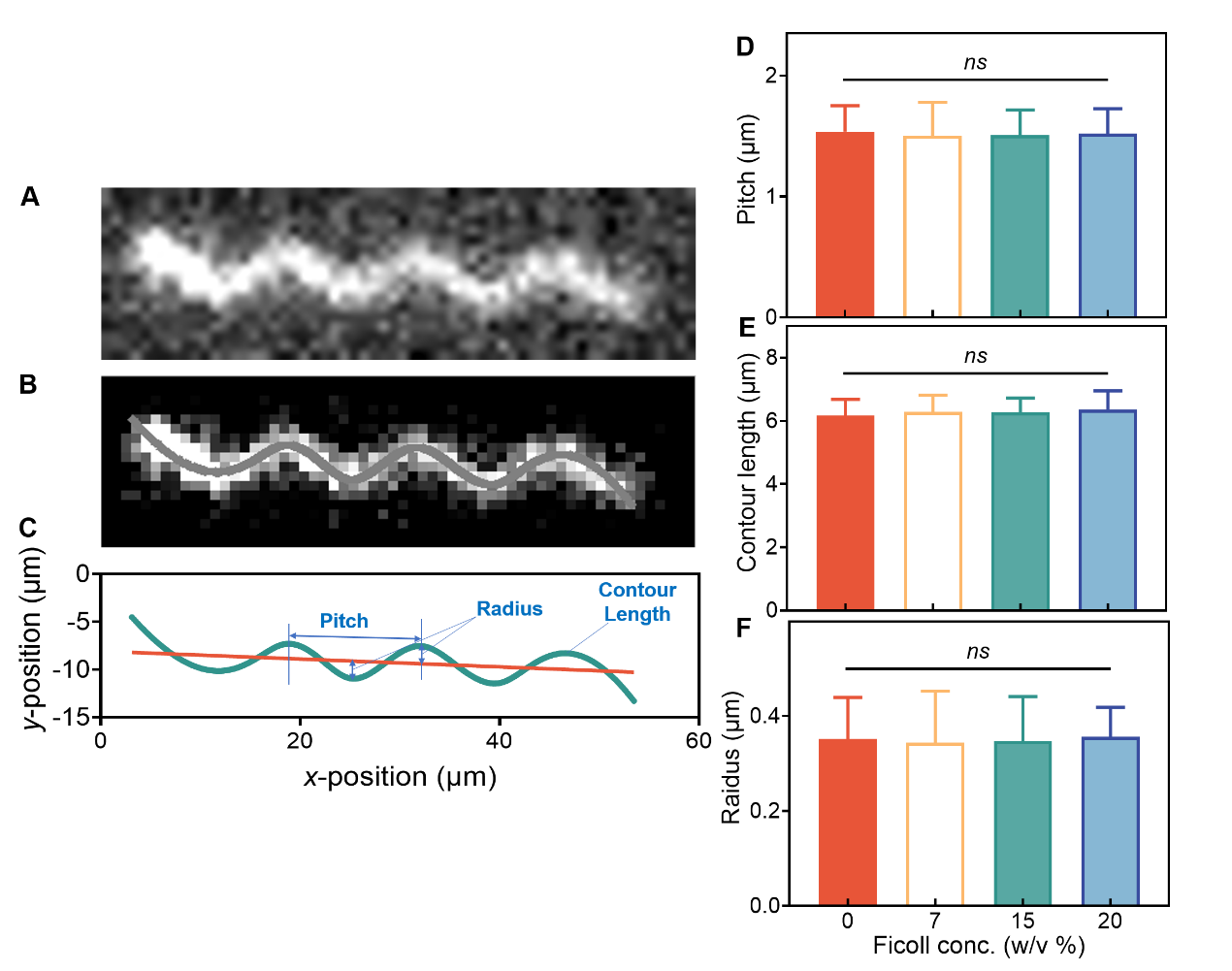


**Fig. S5.** Measurement of the geometrical morphology of the flagellar filament. **A.** The original fluorescence image of a filament. **B.** The image of (A) processed by adjusting image contrast and image binarization with MATLAB. The gray solid line represents a fit with a sinusoidal function for the wave profile of the filament. **C.** The pitch (*p*) and radius (*R*) of a helical filament were obtained from the wave length and amplitude of the fitting. The contour length (*L*) was calculated by using the wave length, amplitude, and waveform number. **D-F.** The results of the pitch (D), contour length (E), and helical radius (F) for the strain MT12 carrying pMT5 in MB mixed with 0%, 7%, 15%, and 20% Ficoll (from left to right). The columns with error bars represent the means and SEMs. “ns” means no statistical difference.


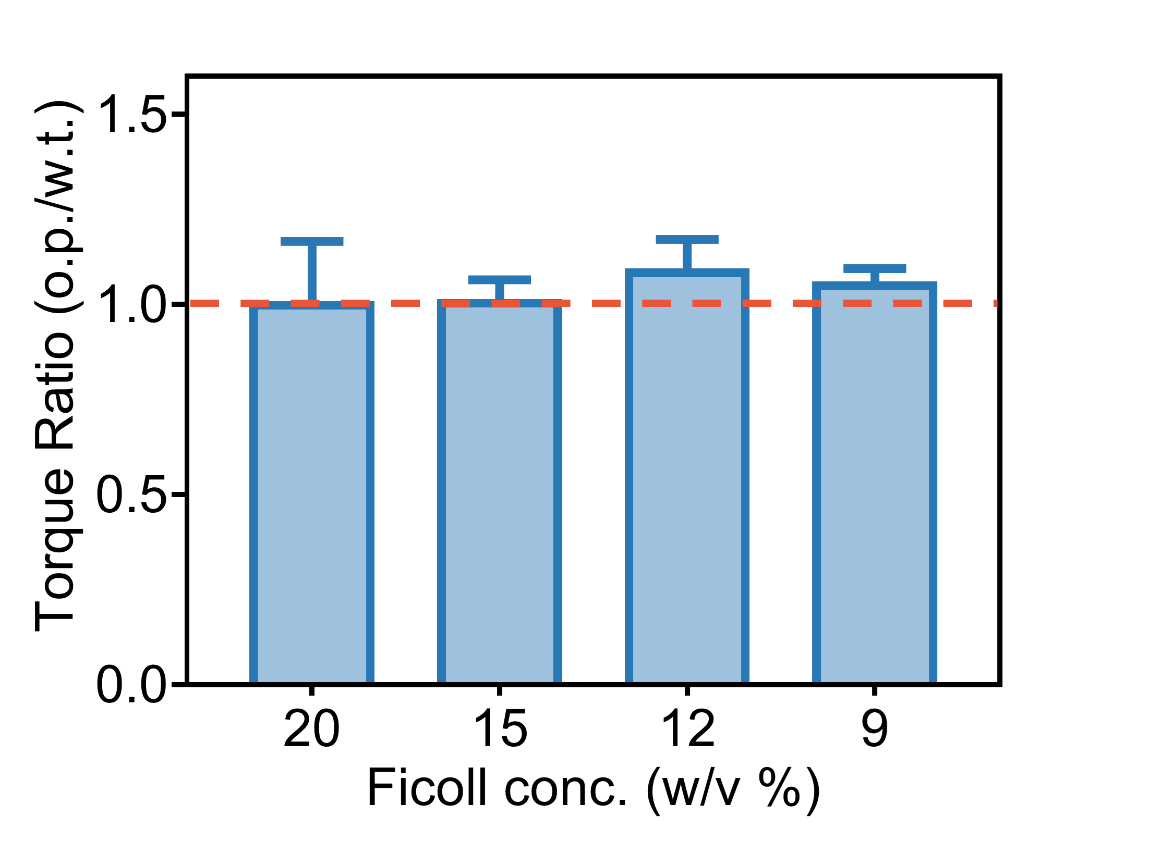


**Fig. S6.** The motor torque ratios (overexpress/wild-type) of Δ*motAB* strain MT20 with *motC*-*motD* gene over expression compared to wild-type expression under high load conditions. Overexpression was achieved by electroporating plasmid pMT7 carrying *motC-motD* genes into MT20 and inducing with 200 μM IPTG. Bars from left to right represent ratios in MB mixed with 20%, 15%, 12%, and 9% (w/v) Ficoll, respectively.


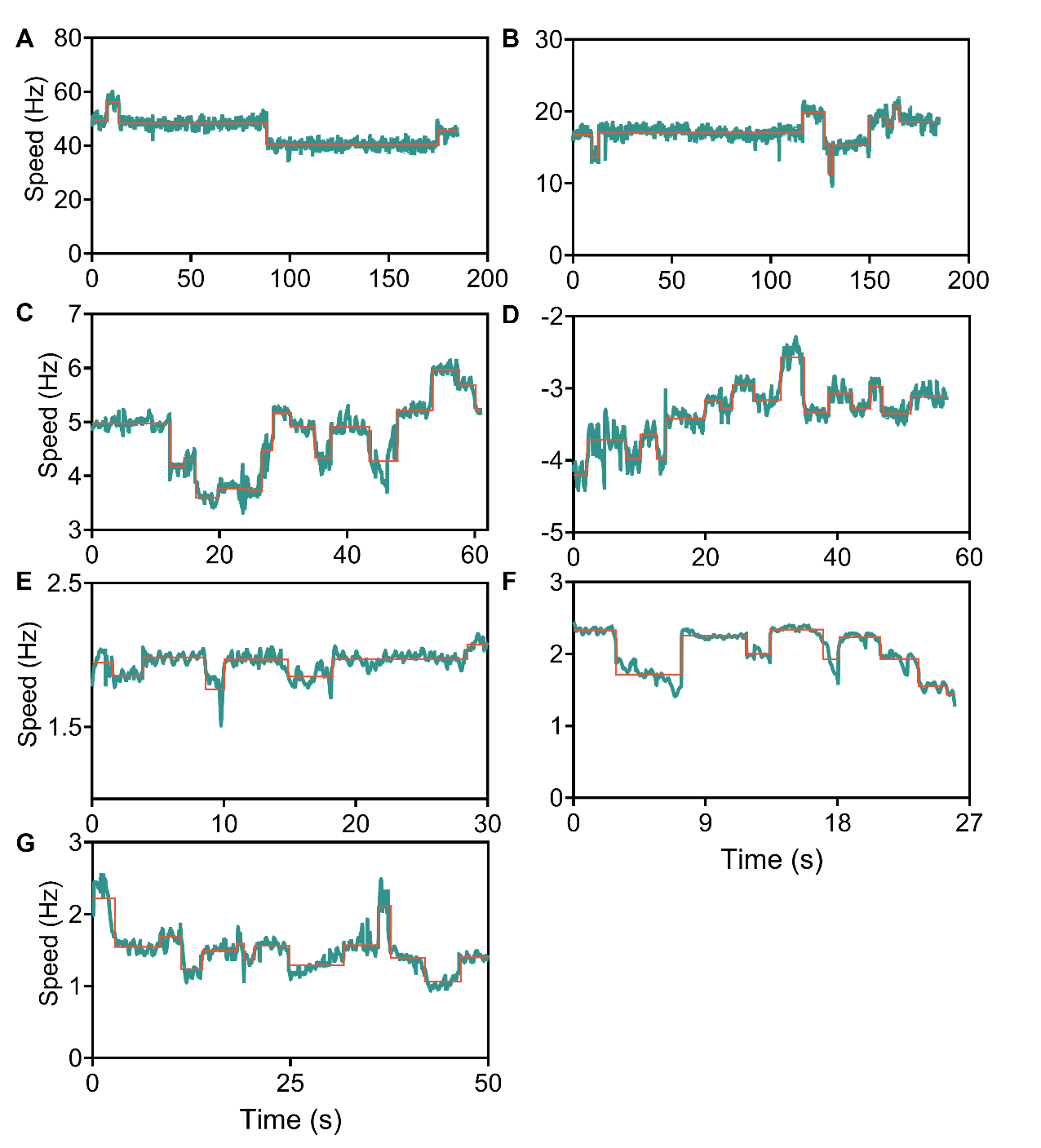
**Fig. S7.** Typical traces of motor speed in a steady state for flagellar motors of the *ΔmotCD* mutant strains. The red solid line represents the average speeds determined by the step-finding algorithm. The trace examples are from: (**A-B**) bead assay in 0% and 2.5% Ficoll, respectively; (**C-G**) tethered-cell assay in 0%, 2%, 3%, 5%, and 7% Ficoll (**C-G**), respectively. Data for panels **A-B** are from a previous study (21).


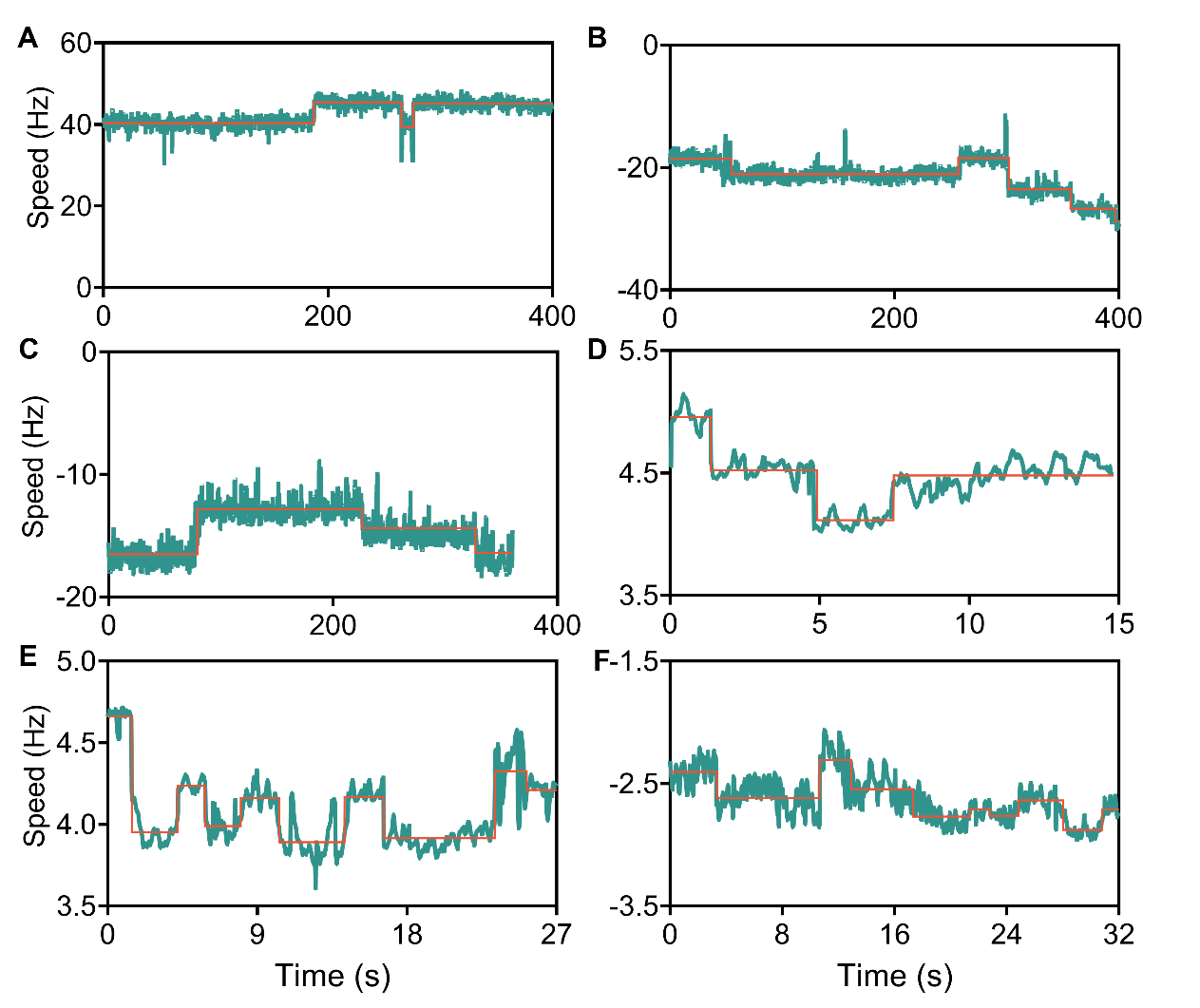


**Fig. S8.** Typical traces of motor speed in a steady state for flagellar motors of the *ΔmotAB* mutant strains. The red solid line represents the average speeds determined by the step-finding algorithm. The trace examples are from: (**A-C**) bead assay in 0%, 5%, and 9% Ficoll, respectively; (**D-F**) tethered-cell assay in 0%, 3%, and 7% Ficoll, respectively. Data for panels **A-B** are from a previous study (21).

*
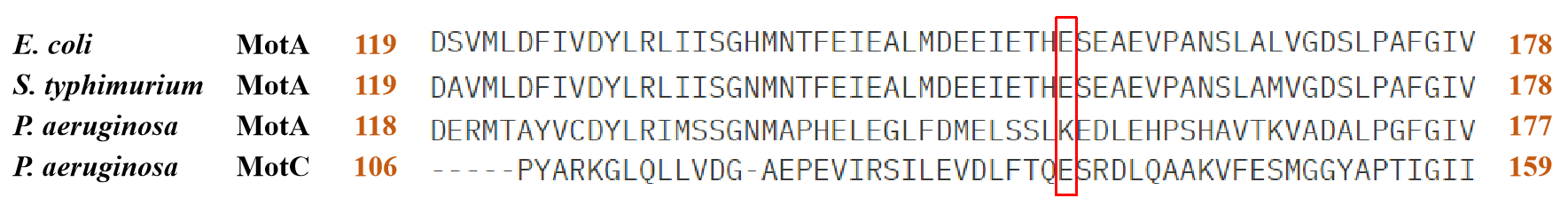
*

**Fig. S9.** Sequence alignment of *P. aeruginosa* MotA (UniProt ID: Q9HUL1) and MotC (G3XD73) with *S. typhimurium* MotA (P55891) and *E. coli* MotA (P09348) to infer their corresponding membrane targeting sequences. The red box highlights the core determining site of the stator autonomous exchange mechanism predicted in previous studies.

**
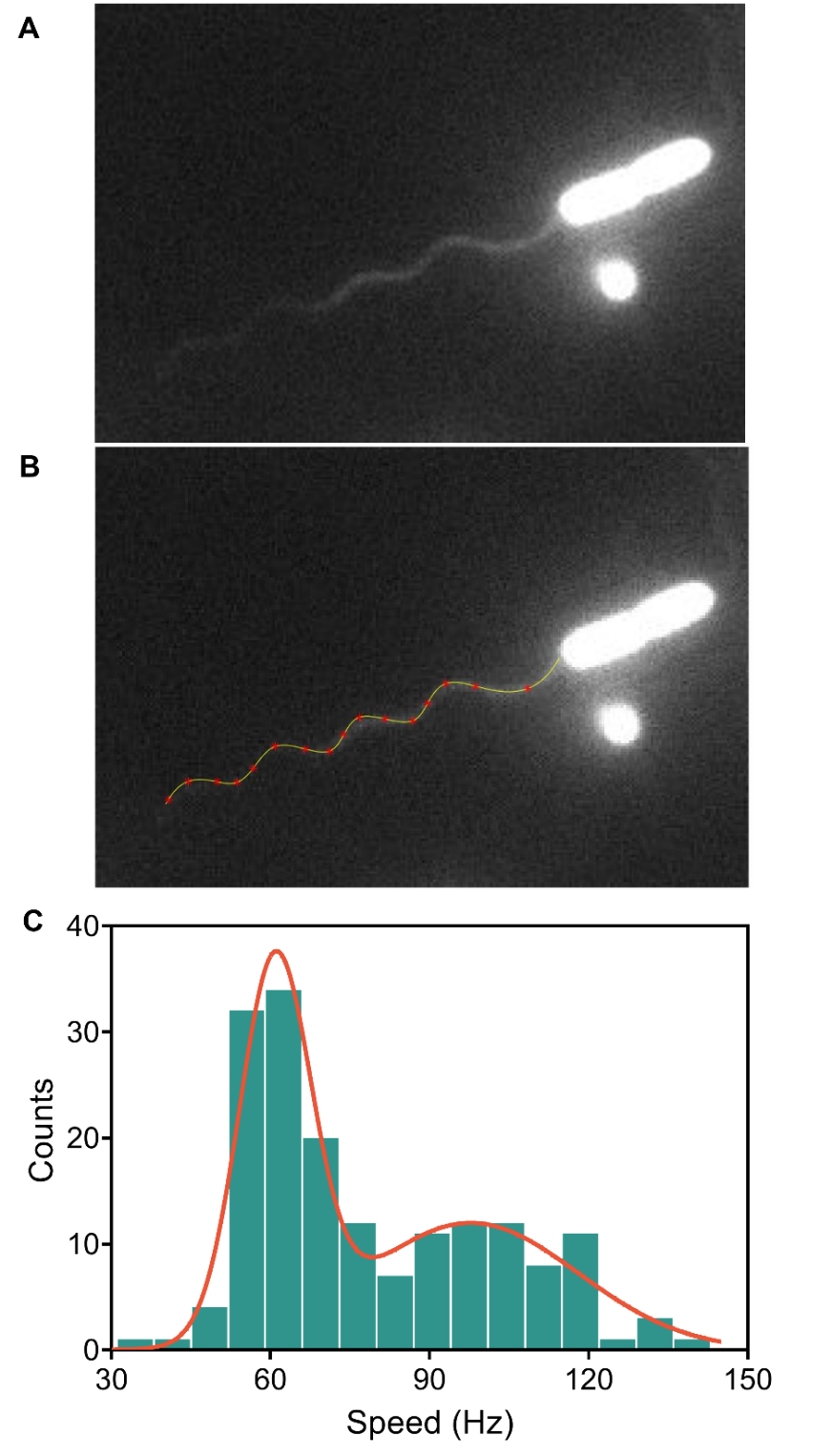
Fig. S10. A.** Fluorescence image of a flagellar filament for *Salmonella* TH14551. **B.** Sinusoidal function fitting curve of the fluorescence image of a filament in (**A)**. The yellow solid line represents a fit with a sinusoidal function for the wave profile of the filament. The red dots indicate the symmetry centers and extreme points of the sinusoidal function. **C.** Distribution of flagellar rotation speeds for *Salmonella* TH14551. The turquoise bars represent the distribution of rotation speeds for individual flagella and flagellar bundles. The red solid line represents the fit using a multimodal Gaussian curve.

**Table S1** Results of the rotational drag coefficients $f_{f}$ for flagellar filament, $f_{motor,t}$ for motors of bacterium cells trapped by optical tweezer, and viscosity $\eta$ of H_2_O medium with different concentrations of Ficoll. The $f_{motor,t}$ value was the mean of calculation results for wild-type, Δ*motCD*, and Δ*motAB* mutant strains under the same loads (mean $\pm$ std).

| **Ficoll conc. (w/v)** | 0% | 2% | 3% | 5% | 7% | 9% | 12% | 15% | 20% |
| --- | --- | --- | --- | --- | --- | --- | --- | --- | --- |
| $\boldsymbol{\eta}$  **(mPa**$\boldsymbol{\cdot}$**s)** | 0.99 | 1.32 | 1.45 | 1.97 | 2.68 | 3.45 | 4.68 | 7.52 | 15.70 |
| $\boldsymbol{f}_{\boldsymbol{f}}$  **(pN**$\boldsymbol{\cdot}$**nm**$\boldsymbol{\cdot}$**s)** | 6.73 | 8.97 | 9.85 | 13.39 | 18.21 | 23.45 | 31.81 | 51.11 | 106.70 |
| $\boldsymbol{f}_{\boldsymbol{motor,t}}$  **(****pN**$\boldsymbol{\cdot}$**nm**$\boldsymbol{\cdot}$**s)** | 6.43 $\pm$0.06 | 8.59 $\pm$0.05 | 9.45 $\pm$0.06 | 12.87 $\pm$0.06 | 17.52 $\pm$0.13 | 22.57 $\pm$0.09 | 30.60 $\pm$0.23 | 48.93 $\pm$0.08 | 100.80 $\pm$0.96 |

**Table S2** Measurement results (mean $\pm$ SEM) and the corresponding number of motors measured for the torque-speed relationship in Fig. 2A.

| **Ficoll conc. (w/v)** | | 0% | 2% | 3% | 5% | 7% | 9% | 12% | 15% | 20% |
| --- | --- | --- | --- | --- | --- | --- | --- | --- | --- | --- |
| **Speed**  **(Hz)** | **wt** | 617.8$\pm15.7$ | 564.0$\pm$24.2 | 544.9$\pm$19.6 | 488.9$\pm$22.7 | 432.1$\pm$25.4 | 380.1$\pm$29.0 | 281.3$\pm$14.6 | 167.8$\pm$10.7 | 72.6 $\pm$5.4 |
|  | **MotAB** | 546.9$\pm14.7$ | 510.2$\pm$12.6 | 480.3$\pm$13.1 | 409.0$\pm$7.7 | 349.1$\pm$8.5 | 269.8$\pm$6.6 | 210.6$\pm$6.7 | 117.2$\pm$4.5 | 28.2 $\pm$3.3 |
|  | **MotCD** | 173.9$\pm$3.4 | 166.1$\pm$3.3 | 160.6$\pm$3.0 | 152.6$\pm$3.0 | 139.0$\pm$3.3 | 133.5$\pm$3.4 | 98.0 $\pm$3.4 | 70.1 $\pm$2.7 | 35.2 $\pm$5.8 |
| **Torque**  **(pN·**  **nm**) | **wt** | 3992  $\pm$106 | 4873  $\pm$217 | 5172  $\pm$197 | 6308  $\pm$307 | 7611  $\pm$469 | 8611  $\pm$690 | 8653  $\pm$470 | 8189  $\pm$549 | 7402  $\pm$576 |
|  | **MotAB** | 3526  $\pm$98 | 4355  $\pm$113 | 4532  $\pm$129 | 5254  $\pm$102 | 6109  $\pm$155 | 6061  $\pm$155 | 6452  $\pm$213 | 5716  $\pm$231 | 2761  $\pm$354 |
|  | **MotCD** | 1103  $\pm$23 | 1420  $\pm$29 | 1504  $\pm$30 | 1950  $\pm$40 | 2410  $\pm$59 | 3004  $\pm$80 | 2966  $\pm$107 | 3428  $\pm$136 | 3556  $\pm$641 |
| **Number of motors** | **wt** | 58 | 42 | 39 | 50 | 49 | 45 | 43 | 75 | 54 |
|  | **MotAB** | 98 | 68 | 57 | 94 | 101 | 86 | 86 | 68 | 35 |
|  | **MotCD** | 101 | 91 | 130 | 106 | 93 | 79 | 78 | 72 | 33 |

**Table S3** Measurement results (mean $\pm$ SEM) and the corresponding number of motors measured for the torque-speed relationships in D_2_O in Fig. 4A-B.

| **Ficoll conc. (w/v)** | | | 0% | 2% | 3% | 5% | 7% | 9% | 12% | 15% | 20% |
| --- | --- | --- | --- | --- | --- | --- | --- | --- | --- | --- | --- |
| **Speed**  **(Hz)** | **MotAB** | 299.4  $\pm$10.9 | | 260.0  $\pm$9.9 | 265.4  $\pm$10.8 | 232.3$\pm$8.1 | 216.8$\pm$9.6 | 169.8$\pm$7.9 | 137.9$\pm$5.2 | 83.0  $\pm$5.4 | 27.9  $\pm$3.5 |
|  | **MotCD** | 83.5  $\pm$3.5 | | 78.8  $\pm$4.8 | 79.2  $\pm$3.8 | 76.4  $\pm$3.8 | 70.2  $\pm$3.9 | 70.5  $\pm$3.0 | 61.0  $\pm$2.8 | 44.3  $\pm$2.8 | 26.9  $\pm$2.2 |
| **Torque**  **(pN·**  **nm)** | **MotAB** | 2345  $\pm$97 | | 2701  $\pm$115 | 3055  $\pm$138 | 3616  $\pm$140 | 4617  $\pm$225 | 4675  $\pm$243 | 5140  $\pm$216 | 4952  $\pm$396 | 3435  $\pm$509 |
|  | **MotCD** | 635  $\pm$29 | | 808  $\pm$58 | 895  $\pm$50 | 1185  $\pm$67 | 1484  $\pm$94 | 1920  $\pm$94 | 2249  $\pm$116 | 2621  $\pm$193 | 3349  $\pm$312 |
| **Number of motors** | **MotAB** | 39 | | 51 | 49 | 49 | 51 | 41 | 47 | 33 | 27 |
|  | **MotCD** | 43 | | 30 | 38 | 35 | 40 | 37 | 41 | 29 | 46 |

**Table S4** Measurement results (mean $\pm$ SEM) of normalized motor speeds and the corresponding number of measured motors for MotAB and MotCD stators under different loads at different pHs in Fig.5A-B.

| **Loads** | | **Low-load** | **Knee-load** | **High-load** |
| --- | --- | --- | --- | --- |
| **MotAB** | **pH=5.7** | 1.07 $\pm$ 0.06 | 0.97 $\pm$ 0.07 | 0.94 $\pm$ 0.08 |
|  | **pH=8.8** | 0.78 $\pm$ 0.03 | 0.75 $\pm$ 0.06 | 0.93 $\pm$ 0.05 |
| **MotCD** | **pH=5.7** | 0.95 $\pm$ 0.03 | 0.90 $\pm$ 0.05 | 0.83 $\pm$ 0.07 |
|  | **pH=8.8** | 0.51 $\pm$ 0.01 | 0.56 $\pm$ 0.03 | 0.77 $\pm$ 0.07 |
| **Number of measured motors** | | **Low-load** | **Knee-load** | **High-load** |
| **MotAB** | **pH=5.7** | 20 | 16 | 26 |
|  | **pH=8.8** | 29 | 18 | 28 |
| **MotCD** | **pH=5.7** | 39 | 30 | 14 |
|  | **pH=8.8** | 37 | 33 | 15 |
